# Perspectives on oral chronic graft-versus-host disease from immunobiology to morbid diagnoses

**DOI:** 10.3389/fimmu.2023.1151493

**Published:** 2023-06-28

**Authors:** Victor Tollemar, Karin Garming Legert, Rachael V. Sugars

**Affiliations:** Division of Oral Diagnostics and Rehabilitation, Department of Dental Medicine, Karolinska Institutet, Stockholm, Sweden

**Keywords:** oral chronic graft-versus-host disease, oral mucosa, oral lichenoid, sicca syndrome, morbid forms, oral potentially malignant disorders, diagnostic criteria, pathophysiology

## Abstract

Chronic Graft-versus-Host Disease (cGVHD) is a major long-term complication, associated with morbidity and mortality in patients following allogenic hematopoietic cell transplantation (HCT) for immune hematopoietic disorders. The mouth is one of the most frequently affected organs after HCT (45-83%) and oral cGVHD, which may appear as the first visible sign. Manifestations present with mucosal lichenoid lesions, salivary gland dysfunction and limited oral aperture. Diagnosis of oral cGVHD severity is based on mucosal lesions with symptoms of sensitivity and pain and reduced oral intake. However, diagnostic difficulties arise due to subjective definitions and low specificity to cover the spectrum of oral cGVHD. In recent years there have been significant improvements in our understanding of the underlying oral cGVHD disease mechanisms. Drawing upon the current knowledge on the pathophysiology and biological phases of oral cGVHD, we address oral mucosa lichenoid and Sjogren’s Syndrome-like sicca syndromes. We consider the response of alloreactive T-cells and macrophages to recipient tissues to drive the pathophysiological reactions and biological phases of acute inflammation (phase 1), chronic inflammation and dysregulated immunity (phase 2), and subsequent aberrant fibrotic healing (phase 3), which in time may be associated with an increased malignant transformation rate. When formulating treatment strategies, the pathophysiological spectrum of cGVHD is patient dependent and not every patient may progress chronologically through the biological stages. As such there remains a need to address and clarify personalized diagnostics and management to improve treatment descriptions. Within this review, we highlight the current state of the art knowledge on oral cGVHD pathophysiology and biological phases. We address knowledge gaps of oral cGVHD, with a view to facilitate clinical management and improve research quality on lichenoid biology and morbid forms of oral cGVHD.

## Introduction

Graft-versus-Host Disease (GVHD) is the major non-relapse related complication following allogenic hematopoietic cell transplantation (HCT) for patients with immune hematopoietic disorders (1). Infused donor cells immunologically target remaining cancer cells by the Graft-versus-Leukemia (GVL) effect (2). However, these cells might also initiate GVHD, where immune competent donor cells respond to the host environment as foreign, leading to inflammation, immune dysfunction, and fibrosis, that often affects multiple organs and tissues (1, 3, 4). GVHD involves different pathophysiological pathways but is broadly described as acute (aGVHD) or chronic (cGVHD) (5). Risks associated with GVHD development include donor sex, age and match, stem cell source, conditioning regime, underlying disease, prior Cytomegalovirus/Epstein Barr viral infections and post-HCT antibody T-cell depletion, as well as cyclophosphamide treatment (6–9). Specific risk factors linked to cGVHD include, prior aGVHD, as well as mismatched or unrelated donor, elderly donor and patient, female donor to male recipient and the use of peripheral blood stem cells (PBSCs) (10).

aGVHD (40-72% patients) is the major short-term cause of morbidity and high risk of mortality. aGVHD typically involves the skin, liver, upper and lower gastrointestinal (GI) tracts (11). The MAGIC consensus guidelines have been developed to support aGVHD clinical staging, and measures the amount of erythematous skin rashes, bilirubin levels and diarrhea (11). Traditionally, aGVHD has been defined with symptoms occurring within the first 100 days after HCT (12, 13). However, the GVHD classifications have been reformulated to, classic aGVHD (≤100 days), persistent late aGVHD (≥100 days), recurrent late aGVHD (new onset >100 days), and aGVHD *de novo* (initiates >100 days) (12).

Approximately 30-70% of patients surviving HCT develop an autoimmune-like inflammation in the form of cGVHD (1, 12).The cGVHD field has been steered by the National Institutes of Health (NIH) Consensus Development Projects in cGVHD (2005, 2014 and 2020), which have focused on diagnostic and staging recommendations to improve clinical trial outcomes (1, 4, 6, 14–22). cGVHD is not only a possible resumption of previous aGVHD (“quiescent” cGVHD onset), cGVHD could onset “*de novo*” (without previous aGVHD). Continuation of acute symptoms into cGVHD is classified as “progressive” cGVHD onset (12). Thus, the NIH Consensus Diagnosis and Staging Working Group proposed cGVHD as an overlap stage, with simultaneous acute and chronic signs, or classic cGVHD with no signs of aGVHD (6, 12). The NIH Consensus global severity scoring involves an eight-item form, that assesses the skin, mouth, eyes, GI tract, liver, lung, joints and fascia, and genital tract system; receiving a score 0 (no/inactive GVHD), score 1 (mild GVHD), score 2 (moderate GVHD), and score 3 (severe GVHD) (4). Additional performance scores are assessed but not incorporated into the severity score (4). This review focuses on the state-of-the-art knowledge on oral cGVHD, our understanding of the pathological and immunological profiles underlying oral cGVHD lichenoid lesions and Sjogren’s Syndrome-like sicca symptoms, and associated disease phases. The review addresses the knowledge gaps to assist personalized management and enhance research quality by expanding our knowledge on lichenoid biology and morbid forms of oral cGVHD.

## Oral cGVHD

Damage to the mucosal barrier and salivary glands may occur during HCT conditioning, and oral complications are common and often related to increased morbidity and decreased quality of life (QoL) (23). Oral aGVHD symptoms seldom occur, unlike manifestations in the gut, where mucositis progresses with typical aGVHD (5, 24). Patients might experience symptoms, such as mucositis, bacterial, *Candida* and viral infections that are attributed to the conditioning regimens before HCT, high-dose immunosuppression post-HCT or the development of early cGVHD (25–29). The oral cavity is one of the most frequently affected organs (45-83%) with cGVHD after HCT using bone marrow stem cells (BMSCs) and particularly after PBSCs (12, 30–32). Oral cGVHD manifestations resemble other autoimmune syndromes within the mouth. Oral lichen planus (OLP) and oral mucosal cGVHD affect the mucosal surfaces with typical white striations, erythema, and ulcerations (4, 31, 33). Salivary gland cGVHD and Sjogren’s Syndrome display sicca-like symptoms and mucoceles, and scleroderma and perioral fibrosis lead to sclerosis and restricted mouth-opening (4, 31, 33). Oral cGVHD is associated with taste dysfunction and masticatory difficulties, mouth pain and sensitivity to food and beverages, which could lead to nutritional deficiencies requiring hospitalization (29, 34–39). These patients can also be sensitive to oral hygiene products and have an increased risk for caries and periodontitis (27, 35, 40).

Despite the early identification of oral cGVHD from the first use of HCT, the field has been hindered by research involving small cohorts with a lack of clear patient descriptions. Initial histopathology studies combined minor salivary gland and oral mucosal features, and the NIH cGVHD Consensus Pathology Working Group (2005) produced a consultation form for pathological evaluation (17, 41, 42). In recent years, our understanding of the pathological conditions involving mucosal and glandular tissues has evolved but the need for better diagnostic and phenotypic criteria remains (33). Bassim and colleagues showed oral cGVHD manifestations as three separate disease presentations, and Cooke et al. (2017) highlighted the diseases biological spectrum (3, 33). These different approaches suggest the need to reconsider our patient classification to one that is focused on individual clinical disease presentations related to histopathological grades, and that reflects the biological phases (33, 43–47). Prolonged severe oral cGVHD has an elevated risk for diminishing QoL and secondary oral malignancies, like oral squamous cell carcinoma (OSCC), can lead to a high risk of mortality (23, 48). There is need to define morbid forms of oral cGVHD associated with decreased QoL and cancer (49).

### Oral cGVHD lichenoid lesion

The NIH Consensus Diagnosis and Staging Working Group (2005 and 2014) defined clinical diagnosis of oral mucosal cGVHD, as lichenoid-like manifestations (4). Gingivitis, mucositis, and erythema are common but not clinically conclusive for either aGVHD or cGVHD and other tests are needed to verify the diagnosis (4). Oral cGVHD lesions are mainly located to the buccal, labial and tongue mucosa (37, 38, 50). Palate lesions are common and probably a sign of extensive oral complications (16, 45, 50). OLP diagnosis includes both clinical and pathological features and is based on a modified World Health Organization criterion (51–54). Clinical features recognize white bilateral papular or reticular striations, with occasional plaque formation, that may be accompanied by erosive lesions, atrophy, and more seldom bullous manifestations (52–54). In the histopathological diagnosis of OLP, a verification of lymphocytic band-like infiltrate with liquefaction degeneration is needed, and the exclusion of dysplasia and verrucous epithelial structures have been suggested (52, 54).

The NIH minimal histopathology criteria for active cGVHD reported clustered to extensive band-like inflammation, and sporadic to widespread exocytosis and apoptosis, which enabled the classification of patients with histopathological stages of “possible” or “likely” oral mucosal cGVHD (14, 43). Lymphocytic exocytosis is a key histological feature in both oral mucosal cGVHD and OLP and was specified in the NIH cGVHD histopathology consultation form as ≥5 cells/10x field of view, as the limit between focal and widespread distribution (14, 17, 43, 54, 55). Originally, epithelial cGVHD damage was described as necrosis, but hydropic degeneration, vacuolization, spongiosis, and squamatization have also been defined (41, 42, 56–58). To align with the OLP criteria, liquefaction degeneration has also been reported in studies, and ranges from sporadic signs of basal cell vacuolization and spongiosis to widespread liquefaction degeneration along the basal cell layer (43, 52). In the most severe cases, complete degeneration of the epithelial connective tissue interface with confluent areas of liquefaction and squamatization have been reported, which is in similarity to skin cGVHD and OLP pathology criteria (14, 43, 52). Programmed cell death, reported as apoptotic-, eosinophilic-, Civatte-bodies or dyskeratotic cells, is assessed by area as ≥1 apoptotic cell/10x field of view by the NIH cGVHD histopathology consultation form (14, 42, 43, 58, 59). However, the extent of apoptosis has been inconsistently reported (56, 58–60). Apoptosis and liquefaction degeneration in OLP have been suggested to be two separate processes of keratinocyte destruction, which could also be reflected in oral mucosal cGVHD (43, 61). Basal membrane alterations including thickening, partial clefts, and Max Joseph separation, have been observed in oral mucosal cGVHD histopathology and clinically erosive OLP (42–44, 56, 58, 62, 63). Flattened rete ridges and atrophic epithelium also appear as common features (41, 43, 64). However, hyperkeratotic or acanthotic oral epithelium has not been reported to define active criteria for oral mucosal cGVHD progression (17, 43). Importantly, a biopsy should be considered to assess distinctive and common manifestations associated with cGVHD, or atypical persistent lesions with increased oral potential malignant risk (14). The histopathological report should verify cGVHD pathology as definitive (likely-cGVHD) or as less evident (possible-cGVHD), based upon the NIH histopathological criteria (14).

### Sjogren’s syndrome-like sicca symptoms

Oral sicca symptoms, such as xerostomia and hyposalivation are common in HCT-patients, and long-term effects could indicate salivary gland cGVHD (39, 65–67). The management and understanding of sicca symptoms post-HCT remain a key knowledge gap, and therefore the field of salivary gland cGVHD lacks validated diagnostic criteria (4, 68). However, studies within the field are impacted by age-related changes in elderly individuals and the use of polypharmacy, which contribute to increased sicca symptoms. Patients receiving radiotherapy for cancer treatment show permanent sicca symptoms, but chemotherapy alone or combined irradiation and chemotherapy conditioning, require further investigations to fully understand the effects (66–69).

Acute and chronic salivary gland GVHD can affect the saliva production in both minor and major glandular structures, but the immunopathology has been principally assessed in the minor glands (46, 66, 67, 70–73) Clinical signs of mucoceles and glandular enlargement, as well as xerostomia are described for both salivary gland cGVHD and Sjogren’s Syndrome, but links between salivary gland cGVHD and overall cGVHD severity remains controversial (16, 35, 66, 71, 74). Patients experiencing salivary gland dysfunction commonly report additional signs associated with a dry mouth, including mucoid viscous saliva, reduced or absent mucosal biofilm, the accumulation of soft debris and erythematous mucosa (75). Supportive information could also measure salivary flow and previous studies have reported <0.2ml/min, although this remains to be verified in consensus studies (33, 47). In comparison, diagnostic criteria for Sjogren’s Syndrome, developed by the American and European Consensus Groups in Rheumatology (2002 and 2016) is based on ≤0.1ml/min unstimulated whole saliva, a Schirmer`s test showing ≤5mm/5min, ocular staining score of ≥5, labial minor salivary gland biopsy with focus score of ≥1/4mm^2^ and autoantibodies against Sjogren’s Syndrome-related antigen A (76, 77).

A labial minor salivary gland biopsy should be used to verify the histopathological criteria of salivary gland cGVHD (14). Histological diagnosis (“possible or likely”) of salivary gland cGVHD has also been based on the NIH cGVHD histopathological consultation form, and the Chisholm-Mason composite score for Sjogren’s Syndrome (14, 17, 46, 47). Periductal and acinar lymphocytic inflammation are considered specific for cGVHD (14). Typically, a mixed infiltrate involving both plasma cells and lymphocytes have been described (14, 46, 47, 56, 58). The presence of Sjogren’s Syndrome-like focused lymphocytic clusters is a contentious issue but salivary gland cGVHD could be related to diffuse peri-ductal and acinar inflammation rather than typical foci, which might depend on disease specificity, severity, and duration post-HCT (46, 47, 56, 65). The NIH cGVHD histopathological consultation form specifically includes exocytosis with periductal and acinar inflammation, in contrast to the Chisholm-Mason composite score (14, 46, 47). Identification of lymphocytic acinar migration with exocytosis is hard to assess and could be attributed to heavy inflammation leading to acinar destruction and difficulties in assessing acinar exocytosis (46). Signs of glandular destruction are a key feature of salivary gland cGVHD, including vacuolization, atrophy, and apoptosis (56, 58, 65). NIH cGVHD histopathological consultation form defines acinar degeneration and apoptosis, whereas others report ductal metaplasia and parenchymal atrophy as signs of destruction (17, 42, 46, 47). In Sjogren’s Syndrome, the description of apoptosis varies considerably, and is typically associated with late stages of the disease (78). Interstitial fibrosis, which commonly occurs in combination with acinar destruction, has been reported to play an important role in the histopathological grade (14, 46, 47, 56). Assessment and interpretation of fibrosis must include the extent and/or extracellular matrix density, as a few signs of fibrosis might indicate a false positive finding (56, 58). However, it is important to consider the influence of conditioning and drug burden that could contribute to tissue destruction and inflammation (14, 65). Minor salivary gland fibrosis is linked to elderly people, and potentially non-specific features following conditioning (14, 47, 65). The issue of salivary gland cGVHD fibrosis warrants further investigation to determine if this feature is due to cGVHD pathogenesis rather than being non-specific or attributed to previous conditioning.

### Perioral fibrosis

A consequence of persistent inflammation in cGVHD is abnormal wound healing, tissue repair and fibrosis, however, the fibrotic pathobiology of cGVHD is poorly described for the oral cavity (3). For a long time, perioral fibrosis was considered as part of skin and oral cGVHD pathophysiology, leading to restricted motion of the oral apparatus (6). However, the 2014 NIH Diagnosis and Staging Working Group revised the clinical criteria for perioral fibrosis to be associated with skin fibrosis, following significant reports where 13% of patients showed both skin sclerosis and limited mouth-opening (4, 14, 33). Symptomatic tightness of the oral mucosa is probably a commonly reported patient feature but associated to both lichenoid inflamed and sclerotic mucous membranes (38). Similar clinical features of lichenoid sclerosus, described in lichenoid skin and vaginal reactions, are uncommon within the oral mucosa (79). Oral fibrosis and sclerosis have been observed within the oral mucosa and salivary gland histopathological profile, but to what extent and functional effect is not fully clear (60, 64, 80). A recent case series reported patients with a history of oral cGVHD to develop oromandibular jaw clenching as bruxism, limited mouth opening and temporomandibular associated symptoms that need further investigations (81).

## Oral cGVHD clinical diagnostic criteria and ancillary measures

**Figure 1:**
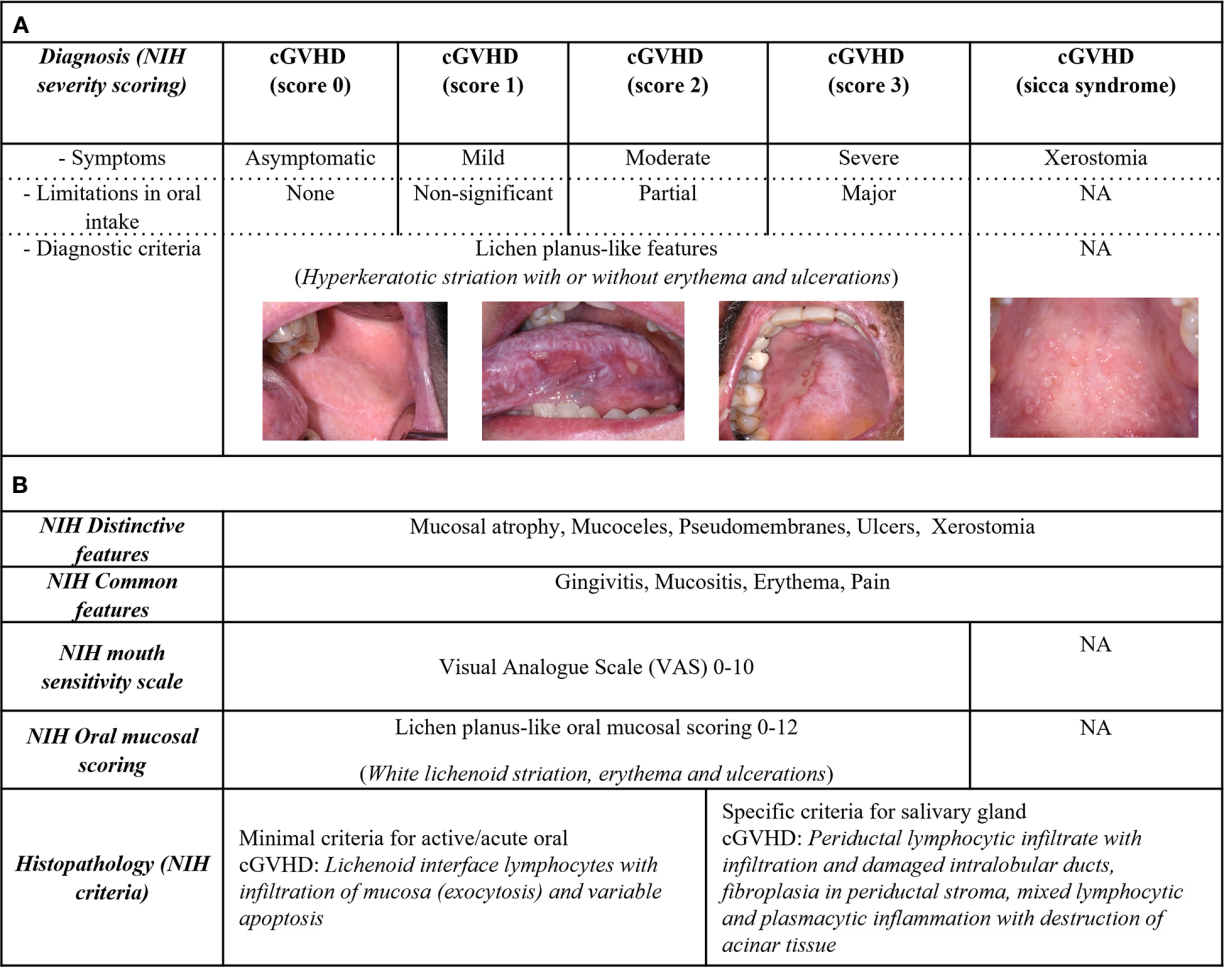
Assessment of oral cGVHD patient characteristics. Oral cGVHD characteristics are described based upon the NIH consensus recommendations. **(A)** Diagnostic features involve lichenoid-like oral mucosal cGVHD, represented by white striations commonly associated with erythema and ulcerations. Severity staging (NIH score 0-3) is based on oral diagnostic clinical signs, sensitivity symptoms (asymptomatic, mild, moderate, or severe) and limitations in oral intake (none, not-significant, partial, or major). Xerostomia is associated with cGVHD sicca-syndrome which lacks diagnostic criteria. **(B)** Distinctive features include mucosal atrophy, pseudomembranous manifestations, ulcers, and xerostomia. Mucoceles are distinctive for cGVHD as both lichenoid- or salivary gland inflammation could be the cause; mucosal atrophy and pseudomembranous manifestations need diagnostic verification. Patient-reported pain, gingivitis, mucositis, and erythema are common signs for cGVHD. The NIH defined cGVHD activity assessment tools in their consensus documents, including a mouth sensitivity scale (0–10) and clinical evaluation for oral mucosal severity scoring (OMRS 0-12). The NIH modified OMRS captures the intensity and extent of erythema lesions (0–3), whereas lichenoid-like manifestations (0–3) and ulcerations (0–6) are graded based on the total area of lesions. The histopathological report should verify cGVHD pathology as definitive (likely-cGVHD) or with less evident (possible-cGVHD) based upon the NIH histopathological criteria.

The NIH organ specific score for oral cGVHD is focused on the mucosal disease manifestations (4). Oral cGVHD severity diagnostic score (**Figure 1A**), is defined by symptoms and limitation of oral intake, ranging from score 0-3. The diagnostic scoring does not include type or distribution of lesion and lacks description of all oral pathophysiology’s including sicca syndrome (4). Studies have commented on the severity scoring to have a low objective value, as clinical interpretation and patient symptoms might differ (31, 45). The NIH cGVHD therapeutic outcome measurements (**Figure 1B**) assess treatment responses and evaluate disease activity, for example the oral mucosal rating scale (OMRS) (16, 82). Patient symptoms for cGVHD activity are captured on a mouth sensitivity scale (Visual Analogue Scale (VAS) 0-10), including irritation from normally tolerated spices, foods, liquids, or flavors (16). The 2005 NIH model for oral lesion scoring (0–15), suggests scores ≥3 as oral cGVHD with 0-2 to be inconclusive (diagnostic global cGVHD score 0) (16, 18, 36, 74, 83). However, clinical improvement or worsening by <3 could be due to inter-rater variability (84). Individual oral mucosal features in the rating scale have also raised concerns, as few patients present with severe ulcerations covering >20% of the oral mucosa (38). The NIH model was refined in 2014 to scoring 0-12, with the removal of mucoceles (16, 18, 74, 83). Mucoceles should only be specific for cGVHD primary sicca-syndrome in the absence of any major lichenoid features (35). cGVHD sicca-syndrome can overlap with oral mucosal cGVHD, but careful consideration is needed as mucoceles might occur secondary to fibrotic lichenoid manifestations or independently (33, 35).

In summary, it is important to appreciate, that diagnostic scores are not recommended to evaluate therapeutic interventions and therefore used as a blunt tool to characterize and compare patients with oral mucosal cGVHD over time (4, 16). It has been acknowledged that many cGVHD patients present with non-active immune cell infiltration and pathology, as well as transforming into malignant conditions with disease progression (43, 44, 48). Thus, it may be proposed that studies should not only generalize oral cGVHD based upon diagnostic score 0-3, as clinical, pathological, and biological status influences the dynamic pathophysiology in the search to define morbid forms of oral cGVHD.

## GVHD pathophysiology and biological phases

**Figure 2:**
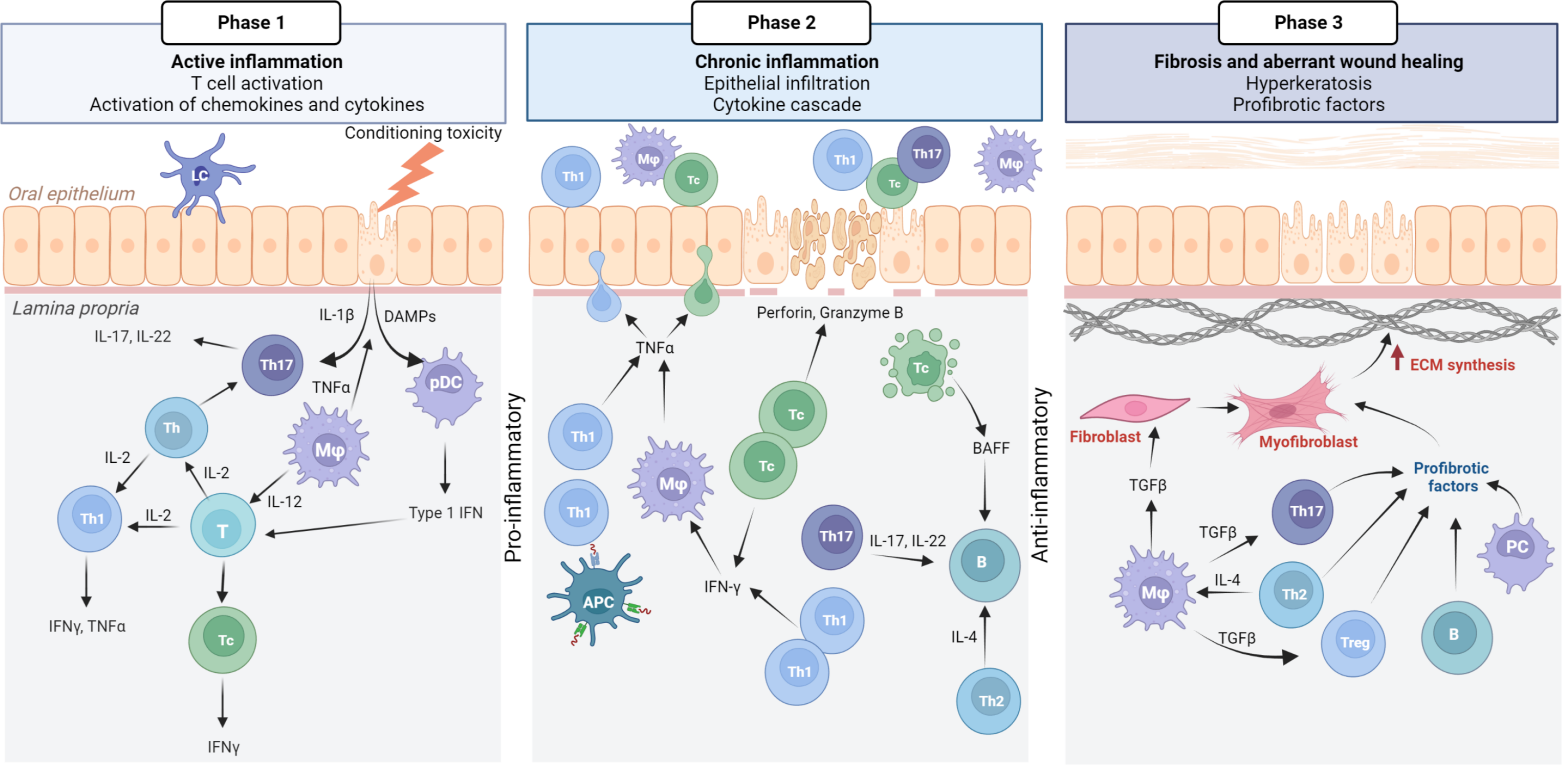
Pathophysiology of oral mucosal GVHD. Active inflammation (phase 1) is initiated by conditioning toxicity, leading to disruption of the mucous membrane, aGVHD and viral reactivation. DAMPs, chemokines, and cytokines are released from endothelial, epithelial, and innate immune cells causing the activation of T cells. Th17 cells initially support epithelial maintenance and acute inflammation. Plasmocytic dendritic cells (pDCs) with type 1 IFN secretion attracts Th1/Tc1 response that drives the chronic inflammatory phase (phase 2). IFNγ secretion promotes activated macrophages (Mφ) with TNFα. Tc cells secrete perforin and granzyme B Tc induced apoptosis leads to the secretion of BAFF, which affects B cells, although this warrants further confirmation in oral mucosal cGVHD pathophysiology. Many cell types, including anti-inflammatory macrophages secrete TGFβ as the inflammatory response switches to a fibrotic stage (phase 3). TGFβ promotes differentiation of Th17 and Treg cells, as well as inducing myofibroblasts and extracellular matrix (ECM) synthesis. Created with BioRender.com.

GVHD pathophysiology is complex, and a three-phase model has evolved that describes GVHD biology (3). The model originates from the GI tract mucosa, where the mucosal barrier is disrupted due to chemotherapy-associated mucositis, however, it can easily be applied to other clinical presentations of cGVHD, such as oral cGVHD (**Figure 2**) (5). In phase 1, the immunocompetent T-cells are known responders to the genetically different human leukocyte antigens (5). Acute inflammation is triggered by the leakage of pathogen-associated molecular patterns, such as lipopolysaccharides, and as a result host tissue damaged-associated molecular patterns are produced, including the proinflammatory cytokines; tumor necrosis factor (TNF)α, interleukins (ILs)-1, -6 and -12 (3, 85). Innate immune cells, such as macrophages and dendritic cells are activated through their Toll-like receptors and migrate to lymph nodes, leading to enhanced antigen presentation and T-cell differentiation (3). An acute immunity cascade is initiated with the activation of naïve T helper (Th)-cells, that polarize and expand into Th1- and Th17-cells, secreting interferon (IFN)γ, IL-2, IL-17 and -22 respectively (5, 85). The paradigm of Th1-/Th2-cell involvement has been discussed in terms of acute/early and chronic/late GVHD pathogenesis, but without consistent data supporting either pathway (3, 86).

Chronic inflammation with increased IFNγ levels, recruit effector Th1-/T cytotoxic (Tc)1-cells into the target tissue, amplifies the cGVHD response in phase 2. Tc cells are the main effectors of cGVHD in the periphery but the coordinated Th-cell, B-cell and macrophage response, with a cytokine cascade, and production of antibodies, remains to be fully understood (87, 88). T regulatory (Treg) cells (FoxP3/CD4+CD25+) function to suppress and control the alloreactive response (3). To add to the complexity, IL-2 activates type 1 and 2 T-cell differentiation and expansion, generating and maintaining Tregs, and inhibits Th17 polarization (5). The IL-2 receptor is also the target for the widely used calcineurin inhibitors (CNIs) (5). A potentially protective role of the phase 2 response includes IFNγ-induced T-cell apoptosis (3, 89). IFNγ further stimulates the production of homeostatic B-cell activation factor (BAFF), which has been reported to be elevated in GVHD patients (3, 90, 91). Increased BAFF levels are associated with delayed B-cell reconstitution and with augmented B-cell receptor signaling and cGVHD severity (90, 92, 93). However, B-cell biology involvement in cGVHD, with associated auto- and alloantibodies, remains unclear but has gained attention over the past decade (87, 91).

Chronic inflammation often results in impaired wound healing, abnormal tissue architecture and dysfunctional fibrosis (3). Thus, phase 3 of GVHD biology is characterized by the activation of extracellular matrix components, typically due to differentiated myofibroblasts leading to the pathogenic stages of fibrosis (3, 31). Transforming growth factor (TGF)β is a hallmark cytokine for the initiation of profibrotic processes and is secreted by many cell types, including tissue macrophages (3, 94). However immune components responsible for sustained fibrosis are not well known. Differentiated B-cells, plasma cells with immunoglobulin (Ig) deposition, as well as Th2-, Th17- and Treg cells are also known to be involved in profibrotic stages, but this is likely to be related to organ-dependent pathways (3, 94). In theory to overcome cGVHD, alloreactive donor T-cells should be depleted, Tregs and thymus function restored (3). Consequently, tissue repair and fibrosis may resolve the progressive GVHD reaction (3).

**Figure 3:**
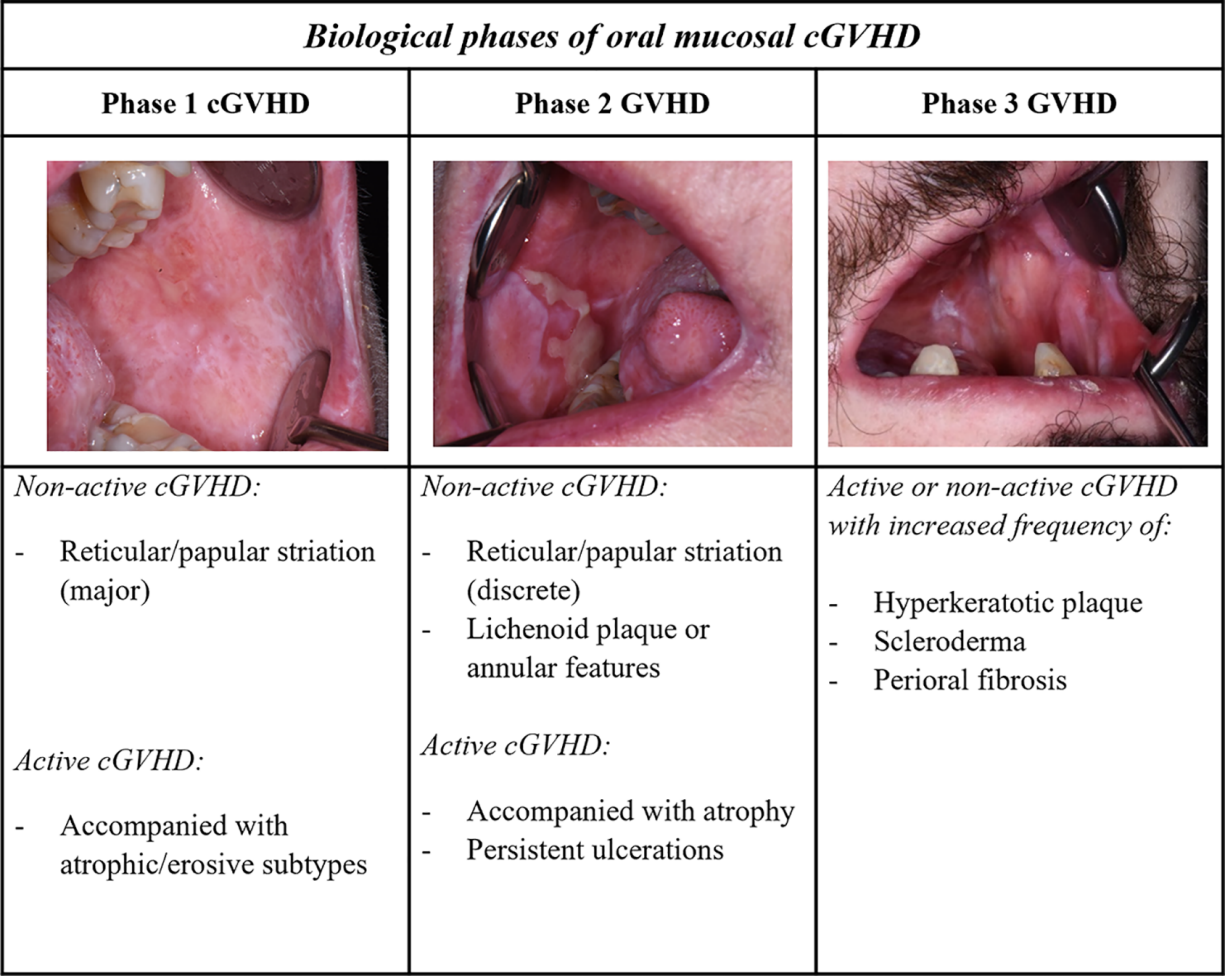
Biological phases of oral mucosal GVHD. A three-phase model described for cGVHD biology can be extrapolated with respect to the oral mucosal presentation. Phases 1 and 2, associated with lichenoid inflammatory components, typically show clinical lichenoid-like signs, whereas phase 3 might be observed with increased white plaque and aberrant fibrotic healing. The complex late phase of GVHD is ascribed with additional scleroderma and perioral fibrosis, as well as increased oral potentially malignant lesions. Different manifestations in the oral mucosa can be observed in both clinically active and non-active cGVHD at each of the different phases.

Emerging evidence highlights that different organ and tissue sites are involved with specific cGVHD pathobiological processes, and that early and late onset involves different pathways (31, 86, 95). Detailed studies are required to investigate the pathophysiological models into type of organ structure; exocrine glandular epithelium with dysfunctional lacrimal- and salivary glands, or manifestations of stratified skin and mucosal epithelium (33). Evidence suggests that organs derived from the same embryologic origin, like ectodermal skin, eyes and oral mucosa share some common cGVHD pathways (33, 96). In the next section, we consider the general cGVHD biological model with respect to our current knowledge and understanding of oral cGVHD pathophysiology (**Figure 3**) (3).

### Pathophysiology and biological phases of oral cGVHD

Immediately post-HCT, white mucosal striations are viewed as non-active clinical features, as lesions often are asymptomatic without any need for treatment, whereas severe lichenoid cGVHD is accompanied by erosive features (**Figures 2**, **3**) (38, 45). Although active inflammatory manifestations (phase 1) and increased pathological features are present for most patients within the first year after HCT, a patient-dependent association needs to be considered (43, 44). Effector mechanisms in oral cGVHD are similar but not identical to patients with OLP or Sjogren’s Syndrome (44, 59, 97–102). Th1, Tc1 and Th17 cells are the predominant cell types in oral mucosal and salivary gland cGVHD, aggregating close to the mucosal epithelium, and within ducts and acini units (3, 44, 46, 59, 97, 103). Dendritic cells have been primarily described as Langerhans cells (CD1a), but evidence of plasmacytoid-like dendritic cell involvement has also been reported in oral cGVHD (44, 46, 59, 60, 98, 100, 104–106). Th1-cells have been suggested to play a role in early phases of the disease but with cGVHD severity these are not as elevated as Tc cells and macrophages (44, 59, 97). Over time in active and severe cGVHD, Th cells stably persistent, whereas Tc cells increase (44). Tc cells in lichenoid oral mucosal cGVHD have been shown to express T-bet, the transcription factor for Th1/Tc1 polarization (59). These type 1 T-cell responses are driven by the IFN cytokines with increased expression of chemokine receptor chemokine receptor (CXCR3) critical for tissue migration (59, 97, 106, 107). The effect of Tc1 cells is distributed via the granzyme-B and perforin pathway (59, 101). Interestingly, post-HCT, unaffected mucosa might display sub-clinical cGVHD, which strongly associates to cGVHD onset and the presence of Th, Tc and Tregs (43, 44, 60, 107–110). Infiltrating T-cells (Th1, Tc1 and Tregs) have been shown to increase in direct proportion to each other but these levels fluctuate with pathological severity and disease duration (44, 58, 59, 97, 107).

Macrophages (CD68 and CD163) in oral mucosal and salivary gland cGVHD tissues are reported inconsistently, but evidence suggests a strong association with oral mucosal cGVHD inflammatory severity and duration (44, 46, 55, 58–60, 100, 111). One study reported CD68+ macrophages to express CD2ap, a plasmacytoid dendritic cell phenotype, and this CD68+CD2ap+ population was associated with oral mucosal cGVHD severity (59). Plasmacytoid dendritic cells have been suggested to migrate into the oral mucosa upon inflammation with type 1 IFN secretion, leading to a storm of chemokines and cytokines, and subsequent cGVHD initiation (59, 106, 112). Macrophages have been described in the mucosa of non-cGVHD patients, as well as those with inactive oral cGVHD pathology, which suggests pro- and anti-inflammatory processes, including phagocytosis and driving the fibrotic processes (3, 44, 113). Hypothetically it could be postulated that host macrophages could have the capacity to limit GVHD and restore conditioning-associated tissue damage, whereas donor macrophages could be involved in GVHD primary inflammatory infiltrate and antigen presenting function and could be used to assist histopathological investigations to understand biological progression (44, 46, 113–115).

During cGVHD propagation into phase 2 (**Figure 2**), persistent clinical ulcerations might be visible due to dysregulated immunity (3). Tc cells seem to diminish, Th cell infiltrate remains frequent, and the macrophage immune profile predominates compared to healthy (44, 59, 94). To our knowledge, no study has examined the characterization of cell populations with respect to the biological phases, but progression into distinctive erosive features have proposed a Th2-polarized response (94, 97). The cytokine profile of Th2 cells are typically IL-4 and IL-5, and C-C chemokine receptor 4 (CCR4), which have been described associated to both oral mucosal and salivary gland cGVHD infiltrations (97). Dendritic cells have been observed in the oral epithelium, and as sporadic migrating cells into the lamina propria of the oral mucosa, however, immunolocalization displayed a patient-dependent variation (44). Studies into Th17 cells are few, but evidence suggests a role in the oral mucosal cGVHD infiltrate (103). Intra-epithelial lymphocytes are even present with reduced inflammation, which suggests persistent effector activity or the involvement of tissue-resident T-cells in the pathogenesis (44, 116). Oral ulcerative cGVHD is often painful but often resolves into the late biological phase (38, 45). A high incidence of clinical hyperkeratotic plaques have been associated with oral cGVHD, and warrants further investigations as potentially the most common oral manifestation late post-HCT (45, 117). We might speculate that cGVHD inflammatory phase 2 displays histopathological and immunological features typical of lichenoid reactions, including lichenoid plaque and annular lesions. However, lichenoid plaques do not significantly reduce when using topical agents, like clobetasol, which suggests more than an active inflammatory etiology (44, 53, 117, 118).

There is a need to define early features of inflammatory salivary gland cGVHD, phases 1 or 2 to identify patients with low saliva flow rate, clinical mucoceles or using biomarkers to improve the diagnosis and management these patients in (21, 72, 102, 119–121). The glandular damage caused by chemotherapy and cGVHD onset has been shown to change saliva composition but not necessarily attributed to decreased salivary flowrate (67). Active oral cGVHD is reported with lower albumin and salivary IgA, and higher complement proteins with altered levels salivary IgG (34, 36, 122). There is a need to determine whether the pathogenesis of Sjogren’s Syndrome differs from salivary gland GVHD (46, 123). Sjogren’s Syndrome infiltrate is described with Th1 cell predominance, but dendritic cells, B-cells and macrophages are also part of the primary response (124). It is interesting that macrophages and CD1a dendritic cells are suggested to increase the focus-score with Sjogren’s Syndrome infiltrate (125, 126). Prolonged salivary gland cGVHD is associated with increased albumin, sodium, and anti-microbial proteins, such as lactoferrin (47, 72, 119, 127). It is noteworthy to recognize that the interaction of and changes to the microbiota in oral cGVHD remains under explored and an area for potential future research (128, 129).

The fibrotic stage (phase 3) is represented as mucosal scleroderma, and salivary gland cGVHD leads to degenerated acinar structures, fibroplasia, and functional impairment of saliva secretion (3, 47). Clinical fibrotic components might be less prominent for oral mucosal cGVHD, but some reports describe oral lichenoid-sclerodermatous plaque and erosions, and oromandibular parafunctions into phase 3 (**Figure 3**) (80, 81). Aberrant healing in phase 3 might represent the transition into non-typical lichenoid clinicopathological features, such as frictional/factitial keratosis, non-reactive or dysplastic leukoplakia (45, 53, 117, 118, 130). A distinct characteristic of the fibrotic biological stage is found in patients with limited mouth opening, displaying features of sclerotic skin cGVHD (**Figure 3**) (3, 33). Within the literature, B-cells are rarely found at sites of oral cGVHD, especially compared to Sjogren’s Syndrome patients, despite increased circulating autoantibodies in cGVHD patients (44, 58, 59, 78, 128). No verified autoantibodies have been correlated with type or severity of cGVHD (47, 128, 131). Mucosal manifestations show aberrant healing properties, genomic instability, and increased potential for hyperkeratotic lesions, proliferative manifestations, and malignant transformation (3, 45, 132–135).

Lichenoid cGVHD immunobiology is complex and might wax and wane with or without therapeutic intervention. A reactivation of GVHD inflammation may happen due to the biological nature of the disease or due to tapering of treatment. Time post HCT might not explain the true nature of cGVHD, and as such the clinicopathological assessment will be important to improve our biological staging of cGVHD. Patients may have progressed to phases 2 or 3, but display active acute or chronic inflammatory components associated with the pathophysiology of phase 1 (4, 45, 130). However, difficulties have arisen in defining the early and late time frames post-HCT, for example, immediate 0-12 months, intermediate 13-47 months and late >47 months (45). The intervention of donor lymphocyte infusion could also trigger a new disease activation, and patients have been reported with both acute oral symptoms and cGVHD features (136). While oral cGVHD duration has been adapted to reflect the biological changes, such as onset 0-3 months, progression 4-6 months, propagation 7-18 months and late >18 months (43). This is an area where the field needs to reflect the pathophysiological phases of clinical lesions and potential biomarkers rather than timepoints. (**Figure 3**). Demarosi et al. reported a patient with classic asymptomatic cGVHD features (phases 1-2) but whose cGVHD reactivated almost four years later with an erythematous patch and overlapping acute, and cGVHD inflammation (137). The lesion later transformed into OSCC. This case study highlights the need for careful consideration of the biology and highlights a morbid proliferative state involving erythematous patches, but these features warrant further investigations (138). In summary, the complex phases of longstanding oral cGVHD (with unconfirmed overlapping biological phases) remains to be explored, including the understanding of the lichenoid biology that might progress and abate leading to manifestations of aberrant healing and dysplasia.

### Morbid cGVHD and considerations for malignant transformation

At the latest NIH Consensus meeting in 2020, skin and fascia, ocular and lung cGVHD were ascribed with high morbidity due to dysfunctional fibrotic pathophysiology (49). Liver and GI tract cGVHD were also identified with a high risk of mortality. Morbid oral cGVHD was discussed in patients, in terms of low QoL and associated increased risk of malignant transformation (49).

Patients with cGVHD experience decreased QoL to varying degrees. Active oral cGVHD contributes to QoL, with functional impairment, activity limitation and pain, regardless of being solely or associated with extra oral cGVHD manifestations (23, 139). Stolze et al. found that diminished QoL, tested with the cGVHD oral health impact profile was correlated with the NIH mouth sensitivity scale (140). The authors further stated that the functional attributes of the oral mucosa and dentition had higher negative impact on the QoL compared to social parameters (140). The association between xerostomia and QoL post allogenic HCT might depend on the extent and cause of salivary gland dysfunction (39, 47, 66, 140). Hyposalivation and dry mouth are not necessarily associated with cGVHD and mucosal involvement (33). However, severe cGVHD sicca syndrome is documented with extensive atypical caries and risk of tooth loss leading to functional impairment (40). Perioral fibrosis may lead to dysfunctional oral aperture, and tentatively decreased QoL. Bassim and colleagues found ≤8% of their cGVHD patients displayed more than one measurable feature of the different pathophysiology’s (oral mucosal, sicca syndrome and perioral fibrosis). The confirmation of more than one pathophysiology including patient associated symptoms, may show significant association with decreased QoL and definable as morbid oral cGVHD (33).

The Oral Potentially Malignant Disorders Working Group (revised consensus 2020) classified lichenoid mucosal manifestations including OLP, oral lichenoid lesions and oral mucosal cGVHD with a verified risk of malignant transformation (48, 53, 141). Other mucosal abnormalities: leukoplakia and its variants (malignant transformation rate ~1–50%), and erythroplakia (malignant transformation rate ~20%) should be considered as high-risk lesions, with the need for personalized management (53, 142–144). The lichenoid field struggles to define if, and what lesions of the lichenoid spectrum should be considered as potentially malignant. Lichenoid malignant transformation rate has often been referred to as low (~1–2%), however a meta-analysis reported that malignant transformation is probably underestimated pin-pointing red atrophic erosive lesions with dysplasia (malignant transformation rate ~6%) carried the highest risks (141). Plaque lesions have also been discussed as potentially high risk, associated with malignant transformation, but difficulties in separating these lesions from leukoplakia have led to conflicting clinical characterizations (53, 145). A case series of oral mucosal cGVHD patients with hyperkeratotic plaques observed that lesions either resolved pontaneously, remained unchanged or progressed to secondary oral cancer over time (130). Another retrospective study found grades of dysplasia, cancer *in situ* and OSCC in oral cGVHD pathology specimens and reported a high prevalence of hyperkeratotic plaque and erosive-atrophic manifestations (146). Oral mucosal cGVHD has been reported with increased genomic alterations leading to molecular abnormalities with multifocal and recurrent OSCC transformation (132, 133, 147). For long-term survivors of oral mucosal cGVHD who continue with immunosuppressive therapy, it is recommended to evaluate manifestations with exophytic, indurated ulcerative associated non-homogenous well-demarcated leukoplakia’s with biopsy (35, 132, 146, 148–151). It is of interest that a Sjogren’s Syndrome diagnosis is also associated with an increased risk of developing secondary lymphoma, something that has not been reported in the field of salivary gland cGVHD (152, 153).

One hindrance to the characterization of oral mucosal cGVHD severity, with associated risk of malignant transformation has been the conflicting terminology (52, 53, 146). To benefit future research advances and increase our understanding of the lichenoid biology with aberrant healing into phase 3, we suggest that lichenoid plaques be considered as striation-like homogenous lesions as they are often surrounded by lichenoid active immunopathological features (53, 118). Thicker and well-demarcated plaques, which are possibly surrounded with erythema, could be considered as an altered sign of aberrant remodeling and verrucous or dysplastic features (4, 53, 118). However, this phenomenon needs close observation as it could be an early sign of cGVHD associated with oral proliferative erythro-leukoplakia (134, 135, 138, 154). These lesions show high malignant transformation and a wide spectrum of histopathological status: corrugated, bulky, verrucous, hyperkeratosis/hyperplasia, or reactive lesions ranging from dysplasia to OSCC (53, 155, 156).

The field of “lichenoid proliferative verrucous leukoplakia” has gained much attention recently following reports describing lichenoid biology progressing into a proliferative state (154, 157–159). OLP white striations might progress and display verrucous plaque with or without dysplasia, and likewise proliferative verrucous leukoplakia might have episodes with biopsy verified OLP inflammation (154, 159). Subsequently, this suggests that oral mucosal cGVHD, or OLP/oral lichenoid lesions-like patients, could within the nature of the disease, “wax and wane” from active to the fibrotic phase, and hypothetically continue into a proliferative state with aberrant tissue remodeling and occasional active inflammatory components (3). This state, with verrucous, erythematous, and probably lichenoid lesions, favors the descriptive condition of proliferative erythro-leukoplakia and needs early recognition in the clinical setting (132, 133, 138). However, longitudinal investigations are needed to describe morbid oral cGVHD with an emphasis on clinical and pathophysiological patient descriptions (48). The oral cavity is a high-malignant transformation risk (potentially ≥10-fold) organ for secondary cancer development post allogenic-HCT (160–162). These patients present with a broad range of oral cGVHD NIH grades (0–3), which suggests the potential need to rephrase the diagnostic nature of asymptomatic non-ulcerative proliferative lesions to highlight morbid oral mucosal cGVHD status (133, 148). Therefore, controlled cohort studies are needed to address which oral cGVHD patients (with characterized diagnostic lesions and score, and designated pathophysiology and biological phase) present with altered morbidity, and to define if manifestations with increased mortality are due to lichenoid cGVHD spectrum or concurrent complications resulting from other allogenic HCT/GVHD associated explanations.

## Oral cGVHD management and treatment options

Management of global cGVHD is based on clinical severity and organ dysfunction. Mild cGVHD is first treated with topical steroids or CNI agents, and systemic corticosteroids are used for patients with moderate to severe cGVHD (163, 164). As such, first line treatments often include a combination of Prednisone with or without CNIs (164, 165). At the recent NIH cGVHD Consensus meeting, a cGVHD treatment report was established (22). As many as 50% of cGVHD patients become steroid refractory and demand a second line treatment within the first two years post-HCT (164, 165). Increased understanding of the different pathophysiology’s involved in cGVHD, has led to multiple trials focused on investigating therapeutics related to specific cGVHD pathways rather than using broad immunosuppressants (22). Many options are available for second line treatments, but no consensus or patient-steered recommendations are available for steroid refractory disease (1). Therapies might involve extracorporeal photopheresis, B-cell depletion (Rituximab), anti-metabolite immunosuppressant (Mycophenolate Mofetil), chemotherapy (Methotrexate), and many other biological drugs are currently being investigated in clinical trials (reviewed in Saidu et al., and Wolff et al) (164–168). In recent years, three drugs were approved by the United States Food and Drug Administration (169). All three (Ibrutinib, Ruxolitinib, Belumosudil) belong to the family of kinase inhibitors and are authorized as second- or third-line treatments for cGVHD (169). Indeed, in an era of personalized medicine, these state-of-the-art immunobiological approaches have demonstrated significant interventional responses for the treatment of oral lichenoid and proliferative lesions, in particular Ruxolitnib (134, 170, 171).

**Figure 4:**
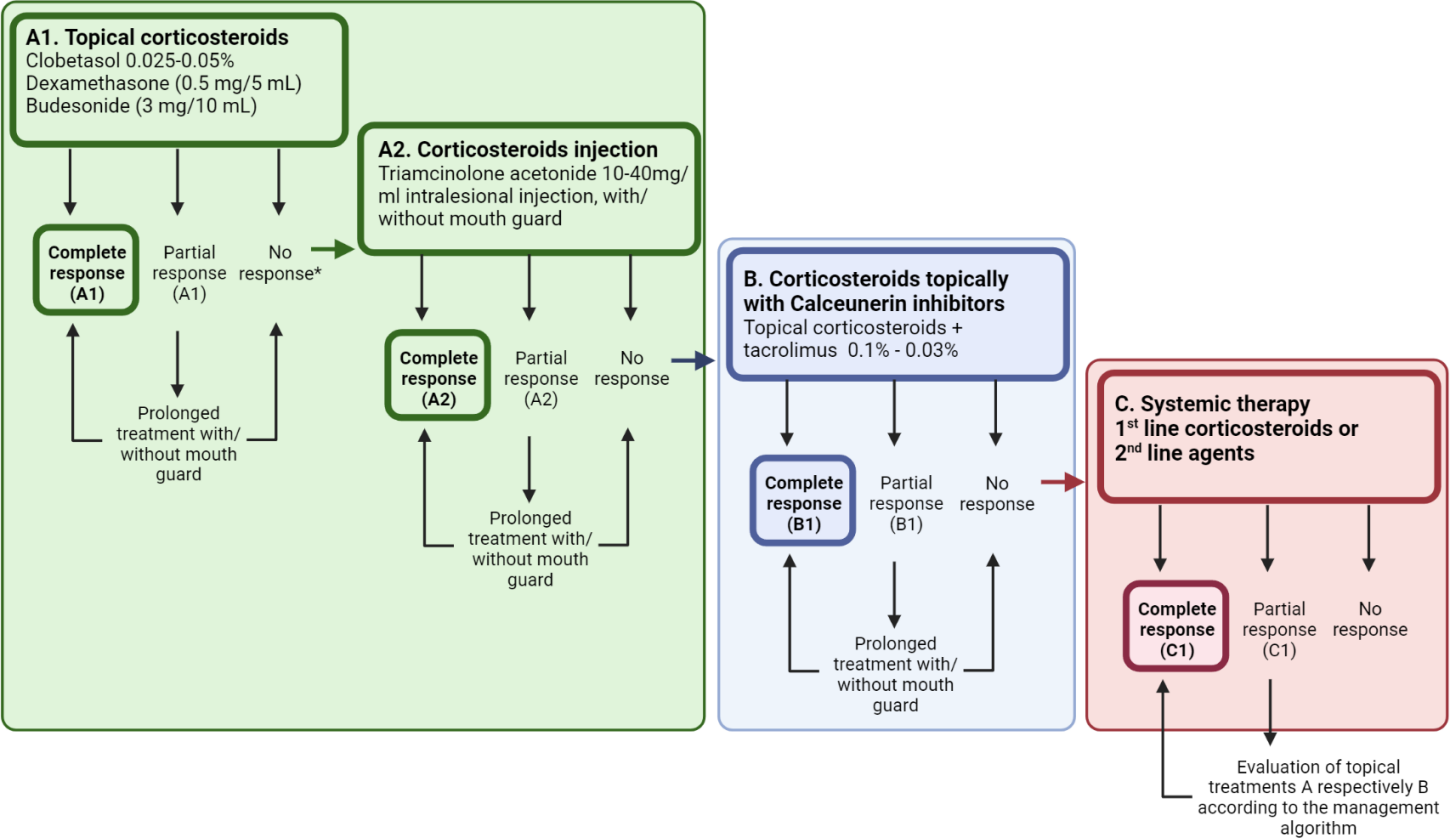
A standardized decision-making treatment algorithm to support step-by-step management of oral cGVHD manifestations. **(A)** Topical or intra-lesional corticosteroids remain the first line therapy, but patients might require additional agents to illicit a response. Therefore, clinicians are encouraged to acknowledge patient subjective responses along with the NIH cGVHD therapeutic measures to assess response and to decide whether to prolong or switch adjunctive treatment in patients with a partial response. **(B, C)** Treatment refractory patients might present at different levels of the decision tree, and continuation of corticosteroids needs consideration, along with the additional effects of calcineurin inhibitors **(B)**, or those demanding systemic treatment or novel second line treatments **(C)** for their oral cGVHD. Created with BioRender.com.

Treatment of oral cGVHD aims to alleviate symptoms and heal ulcerative severe lesions using oral topical ointments or gels of corticosteroids (**Figure 4**) (35). Dexamethasone (0.5 mg/5ml), clobetasol (0.05-0.025%) or triamcinolone 0.1% are often used (35). Only one randomized clinical trial has investigated the superior effect of clobetasol (0.05%), in comparison to dexamethasone for oral mucosal cGVHD, with a significant partial to complete response reported for 85% of the patients (117, 172). Other immunosuppressants, such as topical tacrolimus 0.1%, showed less effective clinical and histopathological responses compared to topical corticosteroids but is preferably used for lip manifestations (35, 57, 60, 117). Evidence points out that combination therapy might give some additional therapeutic effects (57, 173). Topical tacrolimus ointments need to be monitored for altered serum levels, particularly when persisting for longer than two weeks (57). The link between clinical and biological features is needed to guide therapeutic options for the individual cGVHD state (22). With improved patient characterization, standardized clinical decision making for oral cGVHD treatment is an important tool for improved personalized medicine (**Figure 4**). The NIH clinical trial specific core measure OMRS (0–12), and the NIH patient and clinician reported treatment responses (-3 to +3) are vital in clinical research but also for routine care management evaluation (16). Furthermore, the importance of a patient subjective response to treatment was recently highlighted as a concept of QoL, but it did not always cohere with the clinical measurements (174). For oral cGVHD however, a significant association has been reported between NIH clinical and patient reported responses (174).

In addition, long-standing cGVHD and immunosuppressive medications have been shown to raise the risk of *Candida*, bacterial and viral infections, as well as increasing the risks of developing OSCC (29, 35, 48, 175). Oral mucosal cGVHD distinctive signs of atrophy, pseudomembranous and oral ulcers therefore need further testing to rule out concurrent pathologies including possible drug toxicity. Maintenance of oral hygiene for patients with ulcerative and sclerotic mucosa, with decreased mouth-opening is difficult. With the increased occurrence of caries and periodontitis, associated dental treatments are technically harder to perform and expensive for patients (29, 35, 40, 50). Sharp teeth smoothed, and a soft mouth splint manufactured to protect the mucosal surfaces from trauma, particularly if the patients suffer from dry mouth.

Management of dry mouth-related issues include non-prescribed lubricants, to compensate for low saliva function (35). However, there is conflicting evidence as to whether prescribed topical treatments, such as clobetasol, improve the feeling of xerostomia or increase saliva flow rates (117, 173). Sialagogue therapy, commonly Pilocarpine, remains an option but patients need close observation for development of any potential pulmonary side-effects (35, 176). One study tested a soft mouth guard with electrostimulation providing apparent relief of symptoms; however, large-scale studies are needed to evaluate the efficacy (177). In the case of mucoceles, the effect of topical corticosteroids is non-significant, surgical removal or corticosteroid injection might improve clinical status, but this is seldom needed due to spontaneous recovery (35, 173, 178).

Altered perioral fibrosis could lead to limited mouth opening and parafunctions, which affects patients’ oral hygiene but also hinders necessary dental treatment (35, 81). Dental prophylaxis is highly recommended with increased fluoride exposure, close observation, and support from dental professions (35, 81). Some patients might benefit from a jaw trainer, or botulinum toxin injections, to widen and stretch the affected tissues (35, 81). Patients may also feel pain and develop oromandibular and mucosal manifestations related to fibrotic pathobiology, and therefore need to be closely monitored to distinguish from other tentative causalities (35, 81).

With respect to oral cGVHD, severe refractory lesions could also be a target for intra-lesion corticosteroid injection, as well as systemic treatment with prednisolone (35, 179). Novel clinical therapeutics have involved injection of mesenchymal stromal cells for refractory ulcerative oral mucosal cGVHD, topical azathioprine and phototherapy including photobiomodulation or photochemotherapy using psoralen and ultraviolet A have also been explored with positive effects (180–184). In an era of personalized medicine, randomized controlled trials with improved patient characterization are warranted, particularly since biological drug candidates and novel therapeutic interventions are being introduced.

## Discussion and conclusions

Oral cGVHD is a heterogenous disorder affecting both oral mucosal and salivary gland tissue. In times of personalized medicine, tools are required to characterize cGVHD patients for clinical activity, histopathological severity and/or aberrant tissue formation or fibrosis that considers the disease state (3). Treatment strategies are also moving from broad and generalized immunosuppression to targeting disease specific pathways and manifestations (22, 134). Today, diagnosis and management rely on clinical surveillance, that might be accompanied with a biopsy. Oral mucosal cGVHD has been widely studied but the terminology and definitions continuously need refinement to improve future clinical trials (4, 14). Hyperkeratotic plaque is a vague description, and a definitive definition of “lichenoid plaque”, “leukoplakia” or “proliferative (verrucous) leukoplakia” could provide improved support for clinicopathological reports. Likewise, cGVHD severe erythema might represent the high risk erytroplakia lesion in these patients. Oral mucosal cGVHD could also guide the OLP research field with prospective studies to ameliorate our understanding of the potential continuum of lichenoid lesions into a state of proliferative leukoplakia (185). With respect to salivary gland cGVHD, early clinical recognition is complex, and the pathophysiology might only reflect systemic severity or earlier inflammation (14). Therefore, considering potential sub-clinical disease activity (e.g., active disease signs, aberrant tissue remodelling or dysplasia), we emphasize biopsy sampling as a routine recommendation, particularly for patients with prolonged cGVHD (43, 44, 48, 53).

Improved clinical and diagnostic stratification should result in a more homogenous patient population, and the identification of predictive and prognostic biomarkers would provide more diagnostic support (21). Tissue and saliva sampling are vital sources for the identification and validation of biomarkers (21, 120, 186). The pathophysiology has provided key indicators. Th-cells and macrophages are plastic with the ability to polarise into various functionalities and warrant further investigation (44, 187–189). Tc cells as the main drivers of tissue destruction and diagnostic clinical severity, diminish over time but reflect the transition from active inflammation into aberrant tissue remodelling (3, 94) (44). Yet the knowledge gap remains for morbid forms of oral mucosal cGVHD, including the risk for cancer transformation (49). Thus, the exploration of tissue- and saliva-based biomarkers is significantly needed and would add support for clinical care, to understand patients at risk of morbid forms of cGVHD and cancer development.

In conclusion, cGVHD onset is linked to an elevated pathophysiology in healthy and lesion mucosa, as well as in the salivary glands, suggesting systemic measurable activity. Since oral cGVHD continues to be considered as a single composite disorder, discussions concerning the lack of evidence relating to prevalence and impact persist. This is further hindered, in that oral cGVHD lichenoid manifestations are broad with tissue-specific pathways, each with individual clinical subtypes involving active and fibrotic phases, including malignant transformation. Importantly, patients with mild, and severe clinicopathology, present with significantly different immune cell profiles due to pathophysiological differences during onset, progression, propagation, and late stages. Hence, improved clinical and pathological characterization together with assessments in line with biological time-points are needed to improve the outcomes of future research. Acknowledging the obstacles surrounding longstanding oral cGVHD, researchers have a great opportunity to improve the characterization and knowledge surrounding the lichenoid biology leading to morbid forms. As a result, this will lead to an increased understanding of GVHD biology and personalized treatment approaches.

## Author contributions

VT and RS conception of the review, VT, KGL, and RS wrote and reviewed the paper. All authors contributed to the article and approved the submitted version.

## References

[1] Pavletic SZ Martin PJ Schultz KR Lee SJ. The future of chronic graft-Versus-Host disease: introduction to the 2020 national institutes of health consensus development project reports. Transplant Cell Therapy Off Publ Am Soc Transplant Cell Ther (2021) 27(6):448–51. doi: 10.1016/j.jtct.2021.02.034 33785366

[2] Wong E Davis JE Grigg A Szer J Ritchie D. Strategies to enhance the graft versus tumour effect after allogeneic haematopoietic stem cell transplantation. Bone Marrow Transplant. (2019) 54(2):175–89. doi: 10.1038/s41409-018-0244-z 29904127

[3] Cooke KR Luznik L Sarantopoulos S Hakim FT Jagasia M Fowler DH. The biology of chronic graft-versus-Host disease: a task force report from the national institutes of health consensus development project on criteria for clinical trials in chronic graft-versus-Host disease. Biol Blood Marrow Transplant. (2017) 23(2):211–34. doi: 10.1016/j.bbmt.2016.09.023 PMC6020045 27713092

[4] Jagasia MH Greinix HT Arora M Williams KM Wolff D Cowen EW. National institutes of health consensus development project on criteria for clinical trials in chronic graft-versus-Host disease: i. the 2014 diagnosis and staging working group report. Biol Blood Marrow Transplant. (2015) 21(3):389–401.e1. doi: 10.1016/j.bbmt.2014.12.001 25529383 PMC4329079

[5] Ferrara JL Levine JE Reddy P Holler E. Graft-versus-host disease. Lancet. (2009) 373(9674):1550–61. doi: 10.1016/S0140-6736(09)60237-3 PMC2735047 19282026

[6] Filipovich AH Weisdorf D Pavletic S Socie G Wingard JR Lee SJ. National institutes of health consensus development project on criteria for clinical trials in chronic graft-versus-host disease: i. diagnosis and staging working group report. Biol Blood Marrow Transplant. (2005) 11(12):945–56. doi: 10.1016/j.bbmt.2005.09.004 16338616

[7] Carlens S Ringden O Remberger M Lonnqvist B Hagglund H Klaesson S. Risk factors for chronic graft-versus-host disease after bone marrow transplantation: a retrospective single centre analysis. Bone Marrow Transplant. (1998) 22(8):755–61. doi: 10.1038/sj.bmt.1701423 9827972

[8] Keever-Taylor CA Bredeson C Loberiza FR Casper JT Lawton C Rizzo D. Analysis of risk factors for the development of GVHD after T cell-depleted allogeneic BMT: effect of HLA disparity, ABO incompatibility, and method of T-cell depletion. Biol Blood Marrow Transplant. (2001) 7(11):620–30. doi: 10.1053/bbmt.2001.v7.pm11760150 11760150

[9] Lee SJ. New approaches for preventing and treating chronic graft-versus-host disease. Blood. (2005) 105(11):4200–6. doi: 10.1182/blood-2004-10-4023 PMC1895039 15701727

[10] Flowers ME Inamoto Y Carpenter PA Lee SJ Kiem HP Petersdorf EW. Comparative analysis of risk factors for acute graft-versus-host disease and for chronic graft-versus-host disease according to national institutes of health consensus criteria. Blood. (2011) 117(11):3214–9. doi: 10.1182/blood-2010-08-302109 PMC3062319 21263156

[11] Harris AC Young R Devine S Hogan WJ Ayuk F Bunworasate U. International, multicenter standardization of acute graft-versus-Host disease clinical data collection: a report from the mount Sinai acute GVHD international consortium. Biol Blood Marrow Transplant. (2016) 22(1):4–10. doi: 10.1016/j.bbmt.2015.09.001 26386318 PMC4706482

[12] Lee SJ. Classification systems for chronic graft-versus-host disease. Blood. (2017) 129(1):30–7. doi: 10.1182/blood-2016-07-686642 PMC5216262 27821503

[13] Lee SJ Onstad L Chow EJ Shaw BE Jim HSL Syrjala KL. Patient-reported outcomes and health status associated with chronic graft-versus-host disease. Haematologica (2018) 103(9):1535–41. doi: 10.3324/haematol.2018.192930 PMC6119141 29858386

[14] Shulman HM Cardona DM Greenson JK Hingorani S Horn T Huber E. NIH Consensus development project on criteria for clinical trials in chronic graft-versus-host disease: II. the 2014 pathology working group report. Biol Blood Marrow Transplant. (2015) 21(4):589–603. doi: 10.1016/j.bbmt.2014.12.031 25639770 PMC4359636

[15] Martin PJ Schoch G Fisher L Byers V Anasetti C Appelbaum FR. A retrospective analysis of therapy for acute graft-versus-host disease: initial treatment. Blood. (1990) 76(8):1464–72. doi: 10.1182/blood.V76.8.1464.1464 2207321

[16] Lee SJ Wolff D Kitko C Koreth J Inamoto Y Jagasia M. Measuring therapeutic response in chronic graft-versus-host disease. national institutes of health consensus development project on criteria for clinical trials in chronic graft-versus-host disease: IV. the 2014 response criteria working group report. Biol Blood Marrow Transplant. (2015) 21(6):984–99. doi: 10.1016/j.bbmt.2015.02.025 PMC4744804 25796139

[17] Shulman HM Kleiner D Lee SJ Morton T Pavletic SZ Farmer E. Histopathologic diagnosis of chronic graft-versus-host disease: national institutes of health consensus development project on criteria for clinical trials in chronic graft-versus-Host disease: II. pathology working group report. Biol Blood Marrow Transplant. (2006) 12(1):31–47. doi: 10.1016/j.bbmt.2005.10.023 16399567

[18] Pavletic SZ Martin P Lee SJ Mitchell S Jacobsohn D Cowen EW. Measuring therapeutic response in chronic graft-versus-host disease: national institutes of health consensus development project on criteria for clinical trials in chronic graft-versus-Host disease: IV. response criteria working group report. Biol Blood Marrow Transplant. (2006) 12(3):252–66. doi: 10.1016/j.bbmt.2006.01.008 16503494

[19] Pidala J Kitko C Lee SJ Carpenter P Cuvelier GDE Holtan S. National institutes of health consensus development project on criteria for clinical trials in chronic graft-versus-Host disease: IIb. the 2020 preemptive therapy working group report. Transplant Cell Ther (2021) 27(8):632–41. doi: 10.1016/j.jtct.2021.03.029 PMC8934187 33836313

[20] Williams KM Inamoto Y Im A Hamilton B Koreth J Arora M. National institutes of health consensus development project on criteria for clinical trials in chronic graft-versus-Host disease: i. the 2020 etiology and prevention working group report. Transplant Cell Ther (2021) 27(6):452–66. doi: 10.1016/j.jtct.2021.02.035 PMC8217141 33877965

[21] Kitko CL Pidala J Schoemans HM Lawitschka A Flowers ME Cowen EW. National institutes of health consensus development project on criteria for clinical trials in chronic graft-versus-Host disease: IIa. the 2020 clinical implementation and early diagnosis working group report. Transplant Cell Ther (2021) 27(7):545–57. doi: 10.1016/j.jtct.2021.03.033 PMC8803210 33839317

[22] DeFilipp Z Couriel DR Lazaryan A Bhatt VR Buxbaum NP Alousi AM. National institutes of health consensus development project on criteria for clinical trials in chronic graft-versus-Host disease: III. the 2020 treatment of chronic GVHD report. Transplant Cell Ther (2021) 27(9):729–37. doi: 10.1016/j.jtct.2021.05.004 PMC8944207 34147469

[23] DePalo J Chai X Lee SJ Cutler CS Treister N. Assessing the relationship between oral chronic graft-versus-host disease and global measures of quality of life. Oral Oncol (2015) 51(10):944–9. doi: 10.1016/j.oraloncology.2015.07.009 PMC4727395 26277616

[24] Ion D Stevenson K Woo SB Ho VT Soiffer R Antin JH. Characterization of oral involvement in acute graft-versus-host disease. Biol Blood Marrow Transplant. (2014) 20(11):1717–21. doi: 10.1016/j.bbmt.2014.06.031 24979731

[25] Elad S Raber-Durlacher JE Brennan MT Saunders DP Mank AP Zadik Y. Basic oral care for hematology-oncology patients and hematopoietic stem cell transplantation recipients: a position paper from the joint task force of the multinational association of supportive care in Cancer/International society of oral oncology (MASCC/ISOO) and the European society for blood and marrow transplantation (EBMT). Support Care Cancer. (2015) 23(1):223–36. doi: 10.1007/s00520-014-2378-x PMC4328129 25189149

[26] Legert KG Remberger M Ringdén O Heimdahl A Dahllöf G. Reduced intensity conditioning and oral care measures prevent oral mucositis and reduces days of hospitalization in allogeneic stem cell transplantation recipients. Support Care Cancer. (2014) 22(8):2133–40. doi: 10.1007/s00520-014-2190-7 24647488

[27] Heimdahl A Johnson G Danielsson KH Lonqvist B Sundelin P Ringden O. Oral condition of patients with leukemia and severe aplastic anemia. follow-up 1 year after bone marrow transplantation. Oral Surg Oral Med Oral Pathol (1985) 60(5):498–504. doi: 10.1016/0030-4220(85)90238-5 3903599

[28] Bollero P Passarelli PC D'Addona A Pasquantonio G Mancini M Condò R. Oral management of adult patients undergoing hematopoietic stem cell transplantation. Eur Rev Med Pharmacol Sci (2018) 22(4):876–87. doi: 10.26355/eurrev_201802_14365 29509233

[29] Haverman TM Raber-Durlacher JE Rademacher WM Vokurka S Epstein JB Huisman C. Oral complications in hematopoietic stem cell recipients: the role of inflammation. Mediators Inflamm (2014) 2014:378281. doi: 10.1155/2014/378281 24817792 PMC4003795

[30] Flowers ME Parker PM Johnston LJ Matos AV Storer B Bensinger WI. Comparison of chronic graft-versus-host disease after transplantation of peripheral blood stem cells versus bone marrow in allogeneic recipients: long-term follow-up of a randomized trial. Blood. (2002) 100(2):415–9. doi: 10.1182/blood-2002-01-0011 12091330

[31] Fall-Dickson JM Pavletic SZ Mays JW Schubert MM. Oral complications of chronic graft-Versus-Host disease. J Natl Cancer Inst Monogr (2019) 2019(53):lgz007. doi: 10.1093/jncimonographs/lgz007 31425593 PMC6699578

[32] Mays JW Fassil H Edwards DA Pavletic SZ Bassim CW. Oral chronic graft-versus-host disease: current pathogenesis, therapy, and research. Oral Dis (2013) 19(4):327–46. doi: 10.1111/odi.12028 PMC3561479 23107104

[33] Bassim CW Fassil H Mays JW Edwards D Baird K Steinberg SM. Oral disease profiles in chronic graft versus host disease. J Dent Res (2015) 94(4):547–54. doi: 10.1177/0022034515570942 PMC4485216 25740857

[34] Bassim CW Fassil H Mays JW Edwards D Baird K Steinberg SM. Validation of the national institutes of health chronic GVHD oral mucosal score using component-specific measures. Bone Marrow Transplant. (2014) 49(1):116–21. doi: 10.1038/bmt.2013.137 PMC4770537 23995099

[35] Treister N Duncan C Cutler C Lehmann L. How we treat oral chronic graft-versus-host disease. Blood. (2012) 120(17):3407–18. doi: 10.1182/blood-2012-05-393389 22898605

[36] Fassil H Bassim CW Mays J Edwards D Baird K Steinberg SM. Oral chronic graft-vs.-host disease characterization using the NIH scale. J Dent Res (2012) 91(7 Suppl):45S–51S. doi: 10.1177/0022034512450881 22699667 PMC6728450

[37] Piccin A Tagnin M Vecchiato C Al-Khaffaf A Beqiri L Kaiser C. Graft-versus-host disease (GvHD) of the tongue and of the oral cavity: a large retrospective study. Int J Hematol (2018) 108(6):615–21. doi: 10.1007/s12185-018-2520-5 30144000

[38] Treister NS Cook EF Jr. Antin J Lee SJ Soiffer R Woo SB. Clinical evaluation of oral chronic graft-versus-host disease. Biol Blood Marrow Transplant. (2008) 14(1):110–5. doi: 10.1016/j.bbmt.2007.06.017 18158967

[39] Fall-Dickson JM Mitchell SA Marden S Ramsay ES Guadagnini JP Wu T. Oral symptom intensity, health-related quality of life, and correlative salivary cytokines in adult survivors of hematopoietic stem cell transplantation with oral chronic graft-versus-host disease. Biol Blood Marrow Transplant. (2010) 16(7):948–56. doi: 10.1016/j.bbmt.2010.01.017 PMC5443667 20139026

[40] Castellarin P Stevenson K Biasotto M Yuan A Woo SB Treister NS. Extensive dental caries in patients with oral chronic graft-versus-host disease. Biol Blood Marrow Transplant. (2012) 18(10):1573–9. doi: 10.1016/j.bbmt.2012.04.009 22516054

[41] Sale GE Shulman HM Schubert MM Sullivan KM Kopecky KJ Hackman RC. Oral and opthalmic pathology of graft-versus-Host disease in man - predictive value of the lip biopsy. Hum Pathol (1981) 12(11):1022–30. doi: 10.1016/S0046-8177(81)80260-2 7033104

[42] Horn TD Rest EB Mirenski Y Corio RL Zahurak ML Vogelsang GB. The significance of oral mucosal and salivary gland pathology after allogeneic bone marrow transplantation. Arch Dermatol (1995) 131(8):964–5. doi: 10.1001/archderm.1995.01690200104027 7632078

[43] Tollemar V Tudzarovski N Warfvinge G Yarom N Remberger M Heymann R. Histopathological grading of oral mucosal chronic graft-versus-Host disease: Large cohort analysis. Biol Blood Marrow Transplant. (2020) 26(10):1971–9. doi: 10.1016/j.bbmt.2020.06.031 32659433

[44] Tollemar V Strom J Tudzarovski N Habel H Legert KG Heymann R. Immunohistopathology of oral mucosal chronic graft-versus-host disease severity and duration. Oral Dis (2022). doi: 10.1111/odi.14303 35796584

[45] Grein Cavalcanti L Fuentes Araújo RL Bonfim C Torres-Pereira CC. Oral manifestations compatible with chronic graft-versus-Host disease in patients with fanconi anemia. Biol Blood Marrow Transplant. (2015) 21(2):275–80. doi: 10.1016/j.bbmt.2014.10.009 25316110

[46] Tollemar V Arvidsson H Häbel H Tudzarovski N Legert KG Le Blanc K. Grading of minor salivary gland immuno-histopathology post-allogenic hematopoietic cell transplantation. Heliyon (2023) 9(4):e15517. doi: 10.1016/j.heliyon.2023.e15517 37128306 PMC10148098

[47] Imanguli MM Atkinson JC Mitchell SA Avila DN Bishop RJ Cowen EW. Salivary gland involvement in chronic graft-versus-host disease: prevalence, clinical significance, and recommendations for evaluation. Biol Blood Marrow Transplant. (2010) 16(10):1362–9. doi: 10.1016/j.bbmt.2010.03.023 PMC2910178 20353829

[48] Janowiak-Majeranowska A Osowski J. Secondary oral cancer after systemic treatment of hematological malignancies and oral GVHD: a systematic review. Cancers (Basel) (2022) 14(9):2175. doi: 10.3390/cancers14092175 35565303 PMC9102759

[49] Wolff D Radojcic V Lafyatis R Cinar R Rosenstein RK Cowen EW. National institutes of health consensus development project on criteria for clinical trials in chronic graft-versus-Host disease: IV. the 2020 highly morbid forms report. Transplant Cell Ther (2021) 27(10):817–35. doi: 10.1016/j.jtct.2021.06.001 PMC8478861 34217703

[50] Schubert MM Correa ME. Oral graft-versus-host disease. Dent Clin North Am (2008) 52(1):79–109. viii-ix. doi: 10.1016/j.cden.2007.10.004 18154866

[51] Kramer IR Lucas RB Pindborg JJ Sobin LH. Definition of leukoplakia and related lesions: an aid to studies on oral precancer. Oral Surg Oral Med Oral Pathol (1978) 46(4):518–39. 280847

[52] van der Meij EH van der Waal I. Lack of clinicopathologic correlation in the diagnosis of oral lichen planus based on the presently available diagnostic criteria and suggestions for modifications. J Oral Pathol Med (2003) 32(9):507–12. doi: 10.1034/j.1600-0714.2003.00125.x 12969224

[53] Warnakulasuriya S Kujan O Aguirre-Urizar JM Bagan JV González-Moles MÁ Kerr AR. Oral potentially malignant disorders: a consensus report from an international seminar on nomenclature and classification, convened by the WHO collaborating centre for oral cancer. Oral Dis (2021) 27(8):1862–80. doi: 10.1111/odi.13704 33128420

[54] Cheng YS Gould A Kurago Z Fantasia J Muller S. Diagnosis of oral lichen planus: a position paper of the American academy of oral and maxillofacial pathology. Oral surgery Oral medicine Oral Pathol Oral radiology. (2016) 122(3):332–54. doi: 10.1016/j.oooo.2016.05.004 27401683

[55] Soares TC Correa ME Cintra GF Miranda EC Cintra ML. The impact of morphological and immunohistological changes in minor salivary glands on the health of the oral cavity in HSCT patients. Bone Marrow Transplant. (2013) 48(12):1525–9. doi: 10.1038/bmt.2013.105 23892328

[56] Santos PS Coracin FL Barros JC Gallottini MH. Histopathologic diagnosis of chronic graft-versus-host disease of the oral mucosa according to the national institutes of health consensus. Einstein (Sao Paulo). (2014) 12(2):204–10. doi: 10.1590/S1679-45082014AO2974 PMC4891164 25003927

[57] Treister N Li S Kim H Lerman M Sultan A Alyea EP. An open-label phase II randomized trial of topical dexamethasone and tacrolimus solutions for the treatment of oral chronic graft-versus-Host disease. Biol Blood Marrow Transplant. (2016) 22(11):2084–91. doi: 10.1016/j.bbmt.2016.08.020 27590106

[58] Soares AB Faria PR Magna LA Correa ME de Sousa CA Almeida OP. Chronic GVHD in minor salivary glands and oral mucosa: histopathological and immunohistochemical evaluation of 25 patients. J Oral Pathol Med (2005) 34(6):368–73. doi: 10.1111/j.1600-0714.2005.00322.x 15946186

[59] Imanguli MM Swaim WD League SC Gress RE Pavletic SZ Hakim FT. Increased T-bet+ cytotoxic effectors and type I interferon-mediated processes in chronic graft-versus-host disease of the oral mucosa. Blood. (2009) 113(15):3620–30. doi: 10.1182/blood-2008-07-168351 PMC2668847 19168793

[60] Motta A Zhan Q Larson A Lerman M Woo SB Soiffer RJ. Immunohistopathological characterization and the impact of topical immunomodulatory therapy in oral chronic graft-versus-host disease: a pilot study. Oral Dis (2018) 24(4):580–90. doi: 10.1111/odi.12813 PMC5902645 29197137

[61] Bascones-Ilundain C Gonzalez-Moles MA Esparza G Gil-Montoya JA Bascones-Martinez A. Significance of liquefaction degeneration in oral lichen planus: a study of its relationship with apoptosis and cell cycle arrest markers. Clin Exp Dermatol (2007) 32(5):556–63. doi: 10.1111/j.1365-2230.2007.02457.x 17608758

[62] Jungell P Konttinen YT Malmstrom M. Basement membrane changes in oral lichen planus. Proc Finn Dent Soc (1989) 85(2):119–24. 2664770

[63] Jungell P Malmstrom M Wartiovaara J Konttinen Y Sane J. Ultrastructure of oral leukoplakia and lichen planus. i. basal region and inflammatory cells. J Oral Pathol (1987) 16(4):170–8. doi: 10.1111/j.1600-0714.1987.tb02061.x 3114455

[64] Shulman HM Sullivan KM Weiden PL McDonald GB Striker GE Sale GE. Chronic graft-versus-host syndrome in man. a long-term clinicopathologic study of 20 Seattle patients. Am J Med (1980) 69(2):204–17. doi: 10.1016/0002-9343(80)90380-0 6996481

[65] Alborghetti MR Correa ME Adam RL Metze K Coracin FL de Souza CA. Late effects of chronic graft-vs.-host disease in minor salivary glands. J Oral Pathol Med (2005) 34(8):486–93. doi: 10.1111/j.1600-0714.2005.00347.x 16091116

[66] Hull KM Kerridge I Schifter M. Long-term oral complications of allogeneic haematopoietic SCT. Bone Marrow Transplant. (2012) 47(2):265–70. doi: 10.1038/bmt.2011.63 21441960

[67] Boer CC Correa ME Tenuta LM Souza CA Vigorito AC. Post-allogeneic hematopoietic stem cell transplantation (HSCT) changes in inorganic salivary components. Support Care Cancer. (2015) 23(9):2561–7. doi: 10.1007/s00520-015-2613-0 25652148

[68] Jensen SB Pedersen AM Vissink A Andersen E Brown CG Davies AN. A systematic review of salivary gland hypofunction and xerostomia induced by cancer therapies: prevalence, severity and impact on quality of life. Support Care Cancer. (2010) 18(8):1039–60. doi: 10.1007/s00520-010-0827-8 20237805

[69] Chaushu G Itzkovitz-Chaushu S Yefenof E Slavin S Or R Garfunkel AA. A longitudinal follow-up of salivary secretion in bone marrow transplant patients. Oral Surg Oral Med Oral Pathol Oral Radiol Endod. (1995) 79(2):164–9. doi: 10.1016/S1079-2104(05)80276-8 7614178

[70] Nagler R Marmary Y Krausz Y Chisin R Markitziu A Nagler A. Major salivary gland dysfunction in human acute and chronic graft-versus-host disease (GVHD). Bone Marrow Transplant. (1996) 17(2):219–24. 8640170

[71] Drew DZ Donohue T Ramos C Cook L Goodwin R Patronas N. Chronic GVHD manifesting as parotitis after allogeneic hematopoietic SCT. Bone Marrow Transplant. (2009) 44(12):821–2. doi: 10.1038/bmt.2009.79 PMC4214382 19421168

[72] Imanguli MM Atkinson JC Harvey KE Hoehn GT Ryu OH Wu T. Changes in salivary proteome following allogeneic hematopoietic stem cell transplantation. Exp Hematol (2007) 35(2):184–92. doi: 10.1016/j.exphem.2006.10.009 PMC1832107 17258067

[73] Nagler RM Sherman Y Nagler A. Histopathological study of the human submandibular gland in graft versus host disease. J Clin Pathol (1999) 52(5):395–7. doi: 10.1136/jcp.52.5.395 PMC1023082 10560366

[74] Elad S Zeevi I Or R Resnick IB Dray L Shapira MY. Validation of the national institutes of health (NIH) scale for oral chronic graft-versus-host disease (cGVHD). Biol Blood Marrow Transplant. (2010) 16(1):62–9. doi: 10.1016/j.bbmt.2009.08.018 19733252

[75] Carpenter PA Kitko CL Elad S Flowers ME Gea-Banacloche JC Halter JP. National institutes of health consensus development project on criteria for clinical trials in chronic graft-versus-Host disease: v. the 2014 ancillary therapy and supportive care working group report. Biol Blood Marrow Transplant. (2015) 21(7):1167–87. doi: 10.1016/j.bbmt.2015.03.024 PMC4821166 25838185

[76] Shiboski CH Shiboski SC Seror R Criswell LA Labetoulle M Lietman TM. American College of Rheumatology/European league against rheumatism classification criteria for primary sjögren's syndrome: a consensus and data-driven methodology involving three international patient cohorts. Arthritis Rheumatol (Hoboken NJ) (2016) 69(1):35–45. doi: 10.1002/art.39859 PMC5650478 27785888

[77] Vitali C Bombardieri S Jonsson R Moutsopoulos HM Alexander EL Carsons SE. Classification criteria for sjogren's syndrome: a revised version of the European criteria proposed by the American-European consensus group. Ann Rheum Dis (2002) 61(6):554–8. doi: 10.1136/ard.61.6.554 PMC1754137 12006334

[78] Verstappen GM Pringle S. Epithelial-immune cell interplay in primary sjögren syndrome salivary gland pathogenesis. Nat Rev Rheumatol (2021) 17(6):333–48. doi: 10.1038/s41584-021-00605-2 PMC8081003 33911236

[79] Carrozzo M Porter S Mercadante V Fedele S. Oral lichen planus: a disease or a spectrum of tissue reactions? types, causes, diagnostic algorhythms, prognosis, management strategies. Periodontol 2000. (2019) 80(1):105–25. doi: 10.1111/prd.12260 31090143

[80] Şanlı H Akay BN Soydan E Koçyiğit P Arat M İlhan O. Clinical aspects of sclerodermatous type graft-Versus-Host disease after allogeneic hematopoietic cell transplantation. Turkish J haematology Off J Turkish Soc Haematology. (2010) 27(2):91–8. doi: 10.5152/tjh.2010.06 27263450

[81] Joshi P Kiers L Evans A. Oromandibular parafunction in chronic graft-versus-host disease: novel association and treatment approach. Intern Med J (2021) 51(11):1950–3. doi: 10.1111/imj.15569 34796632

[82] Schubert MM Williams BE Lloid ME Donaldson G Chapko MK. Clinical assessment scale for the rating of oral mucosal changes associated with bone marrow transplantation. development of an oral mucositis index. Cancer. (1992) 69(10):2469–77. doi: 10.1002/1097-0142(19920515)69:10<2469::aid-cncr2820691015>3.0.co;2-w 1568168

[83] Treister NS Stevenson K Kim H Woo SB Soiffer R Cutler C. Oral chronic graft-versus-host disease scoring using the NIH consensus criteria. Biol Blood Marrow Transplant. (2010) 16(1):108–14. doi: 10.1016/j.bbmt.2009.09.010 19772943

[84] Mitchell SA Jacobsohn D Thormann Powers KE Carpenter PA Flowers ME Cowen EW. A multicenter pilot evaluation of the national institutes of health chronic graft-versus-host disease (cGVHD) therapeutic response measures: feasibility, interrater reliability, and minimum detectable change. Biol Blood Marrow Transplant. (2011) 17(11):1619–29. doi: 10.1016/j.bbmt.2011.04.002 PMC3158826 21536143

[85] Zeiser R Blazar BR. Acute graft-versus-Host disease - biologic process, prevention, and therapy. N Engl J Med (2017) 377(22):2167–79. doi: 10.1056/NEJMra1609337 PMC6034180 29171820

[86] Rozmus J Schultz KR Wynne K Kariminia A Satyanarayana P Krailo M. Early and late extensive chronic graft-versus-host disease in children is characterized by different Th1/Th2 cytokine profiles: findings of the children's oncology group study ASCT0031. Biol Blood marrow Transplant (2011) 17(12):1804–13. doi: 10.1016/j.bbmt.2011.05.011 PMC3190042 21669298

[87] Sarantopoulos S Blazar BR Cutler C Ritz J. B cells in chronic graft-versus-host disease. Biol Blood Marrow Transplant. (2015) 21(1):16–23. doi: 10.1016/j.bbmt.2014.10.029 25452031 PMC4295503

[88] Podgorny PJ Liu Y Dharmani-Khan P Pratt LM Jamani K Luider J. Immune cell subset counts associated with graft-versus-host disease. Biol Blood Marrow Transplant. (2014) 20(4):450–62. doi: 10.1016/j.bbmt.2014.01.002 24406506

[89] Reddy P Teshima T Kukuruga M Ordemann R Liu C Lowler K. Interleukin-18 regulates acute graft-versus-host disease by enhancing fas-mediated donor T cell apoptosis. J Exp Med (2001) 194(10):1433–40. doi: 10.1084/jem.194.10.1433 PMC2193680 11714750

[90] Jacobson CA Sun L Kim HT McDonough SM Reynolds CG Schowalter M. Post-transplantation b cell activating factor and b cell recovery before onset of chronic graft-versus-host disease. Biol Blood Marrow Transplant. (2014) 20(5):668–75. doi: 10.1016/j.bbmt.2014.01.021 PMC3985384 24462743

[91] Stikvoort A Chen Y Rådestad E Törlén J Lakshmikanth T Björklund A. Combining flow and mass cytometry in the search for biomarkers in chronic graft-versus-Host disease. Front Immunol (2017) 8:717. doi: 10.3389/fimmu.2017.00717 28674539 PMC5474470

[92] Jia W Poe JC. BAFF promotes heightened BCR responsiveness and manifestations of chronic GVHD after allogeneic stem cell transplantation. Blood (2021) 137(18):2544–57. doi: 10.1182/blood.2020008040 PMC8109011 33534893

[93] Sarantopoulos S Stevenson KE Kim HT Bhuiya NS Cutler CS Soiffer RJ. High levels of b-cell activating factor in patients with active chronic graft-versus-host disease. Clin Cancer Res (2007) 13(20):6107–14. doi: 10.1158/1078-0432.CCR-07-1290 PMC2941091 17947475

[94] Zeiser R Blazar BR. Pathophysiology of chronic graft-versus-Host disease and therapeutic targets. N Engl J Med (2017) 377(26):2565–79. doi: 10.1056/NEJMra1703472 29281578

[95] Kim D Won HH Su S Cheng L Xu W Hamad N. Risk stratification of organ-specific GVHD can be improved by single-nucleotide polymorphism-based risk models. Bone Marrow Transplant. (2014) 49(5):649–56. doi: 10.1038/bmt.2014.20 24583628

[96] Jeppesen H Gjærde LK Lindegaard J Julian HO Heegaard S Sengeløv H. Ocular chronic graft-versus-Host disease and its relation to other organ manifestations and outcomes after allogeneic hematopoietic cell transplantation. Transplant Cell Ther (2022) 28(12):833.e1–.e7. doi: 10.1016/j.jtct.2022.08.016 36002105

[97] Hayashida JN Nakamura S Toyoshima T Moriyama M Sasaki M Kawamura E. Possible involvement of cytokines, chemokines and chemokine receptors in the initiation and progression of chronic GVHD. Bone Marrow Transplant. (2013) 48(1):115–23. doi: 10.1038/bmt.2012.100 22659679

[98] Hasseus B Jontell M Brune M Johansson P Dahlgren UI. Langerhans cells and T cells in oral graft versus host disease and oral lichen planus. Scand J Immunol (2001) 54(5):516–24. doi: 10.1046/j.1365-3083.2001.00988.x 11696204

[99] Nakamura S Hiroki A Shinohara M Gondo H Ohyama Y Mouri T. Oral involvement in chronic graft-versus-host disease after allogeneic bone marrow transplantation. Oral Surg Oral Med Oral Pathol Oral Radiol Endod. (1996) 82(5):556–63. doi: 10.1016/S1079-2104(96)80203-4 8936521

[100] Sato M Tokuda N Fukumoto T Mano T Sato T Ueyama Y. Immunohistopathological study of the oral lichenoid lesions of chronic GVHD. J Oral Pathol Med (2006) 35(1):33–6. doi: 10.1111/j.1600-0714.2005.00372.x 16393251

[101] Pimentel VN de Matos LS Soares TC Adam R Metze K Correa ME. Perforin and granzyme b involvement in oral lesions of lichen planus and chronic GVHD. J Oral Pathol Med (2010) 39(10):741–6. doi: 10.1111/j.1600-0714.2010.00917.x 20618609

[102] Nagler RM Nagler A. The molecular basis of salivary gland involvement in graft–vs.–host disease. J Dent Res (2004) 83(2):98–103. doi: 10.1177/154405910408300203 14742644

[103] Klimczak A Suchnicki K Sedzimirska M Lange A. Diverse activity of IL-17+ cells in chronic skin and mucosa graft-Versus-Host disease. Arch Immunol Ther Exp (Warsz). (2019) 67(5):311–23. doi: 10.1007/s00005-019-00549-2 PMC6732123 31177288

[104] Fujii H Ohashi M Nagura H. Immunohistochemical analysis of oral lichen-planus-like eruption in graft-versus-host disease after allogeneic bone marrow transplantation. Am J Clin Pathol (1988) 89(2):177–86. doi: 10.1093/ajcp/89.2.177 3277380

[105] Botari CM Nunes AJ Souza MP Orti-Raduan ES Salvio AG. Oral chronic graft-versus-host disease: analysis of dendritic cells subpopulations. Bras Dermatol (2014) 89(4):632–7. doi: 10.1590/abd1806-4841.20142464 PMC4148278 25054751

[106] Hakim FT Memon S. Upregulation of IFN-inducible and damage-response pathways in chronic graft-versus-Host disease. J Immunol (2016) 197(9):3490–503. doi: 10.4049/jimmunol.1601054 PMC5101132 27694491

[107] Imanguli MM Cowen EW Rose J Dhamala S Swaim W Lafond S. Comparative analysis of FoxP3 regulatory T cells in the target tissues and blood in chronic graft versus host disease. Leukemia. (2014) 28(10):2016–27. doi: 10.1038/leu.2014.92 PMC7670308 24577531

[108] Resende RG Correia-Silva JD Arao TC Brito JAR Bittencourt H Gomez RS. Oral cGVHD screening tests in the diagnosis of systemic chronic graft-versus-host disease. Clin Oral Investig (2012) 16(2):565–70. doi: 10.1007/s00784-011-0529-8 21369795

[109] Prochorec-Sobieszek M Nasilowska-Adamska B Szumera-Cieckiewicz A Tomaszewska A Halaburda K Szczepinski A. The significance of oral labial biopsy in hepatic graft-versus-host disease diagnosis in patients following allogeneic hematopoietic stem cell transplantation–a preliminary report. Ann Transplant. (2012) 17(3):85–92. doi: 10.12659/aot.883462 23018260

[110] Vassallo C Brazzelli V Alessandrino PE Varettoni M Ardigo M Lazzarino M. Normal-looking skin in oncohaematological patients after allogenic bone marrow transplantation is not normal. Br J Dermatol (2004) 151(3):579–86. doi: 10.1111/j.1365-2133.2004.06072.x 15377343

[111] Mattsson T Sundqvist KG Heimdahl A Dahllof G Ljungman P Ringden O. A comparative immunological analysis of the oral mucosa in chronic graft-versus-host disease and oral lichen planus. Arch Oral Biol (1992) 37(7):539–47. doi: 10.1016/0003-9969(92)90136-V 1359859

[112] Hovav AH. Dendritic cells of the oral mucosa. Mucosal Immunol (2014) 7(1):27–37. doi: 10.1038/mi.2013.42 23757304

[113] van Balen P van der Zouwen B Kruisselbrink AB Eefting M Szuhai K Jordanova ES. Tissue damage caused by myeloablative, but not non-myeloablative, conditioning before allogeneic stem cell transplantation results in dermal macrophage recruitment without active T-cell interaction. Front Immunol (2018) 9:331. doi: 10.3389/fimmu.2018.00331 29535719 PMC5835032

[114] Hashimoto D Chow A Greter M Saenger Y Kwan W-H Leboeuf M. Pretransplant CSF-1 therapy expands recipient macrophages and ameliorates GVHD after allogeneic hematopoietic cell transplantation. J Exp Med (2011) 208(5):1069–82. doi: 10.1084/jem.20101709 PMC3092347 21536742

[115] Jardine L Cytlak U Gunawan M Reynolds G Green K Wang XN. Donor monocyte-derived macrophages promote human acute graft-versus-host disease. J Clin Invest. (2020) 130(9):4574–86. doi: 10.1172/JCI133909 PMC7456218 32453711

[116] Jiang H Fu D Bidgoli A Paczesny S. T Cell subsets in graft versus host disease and graft versus tumor. Front Immunol (2021) 12. doi: 10.3389/fimmu.2021.761448 PMC8525316 34675938

[117] Noce CW Gomes A Shcaira V Corrêa ME Moreira MC Silva Júnior A. Randomized double-blind clinical trial comparing clobetasol and dexamethasone for the topical treatment of symptomatic oral chronic graft-versus-host disease. Biol Blood Marrow Transplant. (2014) 20(8):1163–8. doi: 10.1016/j.bbmt.2014.04.009 24727333

[118] Woo SB. Oral epithelial dysplasia and premalignancy. Head Neck Pathol (2019) 13(3):423–39. doi: 10.1007/s12105-019-01020-6 PMC6684678 30887394

[119] Izutsu KT Schubert MM Truelove EL Shulman HM Sale GE Morton TH. The predictive value of elevated labial saliva sodium concentration: its relation to labial gland pathology in bone marrow transplant recipients. Hum Pathol (1983) 14(1):29–35. doi: 10.1016/S0046-8177(83)80043-4 6339354

[120] Costa-da-Silva AC Aure MH Dodge J Martin D Dhamala S Cho M. Salivary ZG16B expression loss follows exocrine gland dysfunction related to oral chronic graft-versus-host disease. iScience. (2022) 25(1):103592. doi: 10.1016/j.isci.2021.103592 35005541 PMC8718990

[121] Souza MM de Paula FM Hsieh R Macedo MC Corral MA Nunes TB. Could mucin 16 and colony-stimulating factor 2-receptor beta possible graft versus host disease biomarkers? medical hypotheses. Med Hypotheses. (2017) 100:89–93. doi: 10.1016/j.mehy.2017.01.017 28236856

[122] Grkovic L Baird K Steinberg SM Williams KM Pulanic D Cowen EW. Clinical laboratory markers of inflammation as determinants of chronic graft-versus-host disease activity and NIH global severity. Leukemia. (2012) 26(4):633–43. doi: 10.1038/leu.2011.254 PMC3262945 22005783

[123] Hiroki A Nakamura S Shinohara M Gondo H Ohyama Y Hayashi S. A comparison of glandular involvement between chronic graft-versus-host disease and sjögren's syndrome. Int J Oral Maxillofac Surg (1996) 25(4):298–307. doi: 10.1016/S0901-5027(06)80062-7 8910118

[124] Parisis D Chivasso C Perret J Soyfoo MS Delporte C. Current state of knowledge on primary sjögren’s syndrome, an autoimmune exocrinopathy. J Clin Med (2020) 9(7):2299. doi: 10.3390/jcm9072299 32698400 PMC7408693

[125] van Blokland SCA Wierenga-Wolf AF van Helden-Meeuwsen CG Drexhage HA Hooijkaas H van de Merwe JP. Professional antigen presenting cells in minor salivary glands in sjögren’s syndrome: potential contribution to the histopathological diagnosis? Lab Invest (2000) 80(12):1935–41. doi: 10.1038/labinvest.3780203 11140705

[126] Manoussakis MN Boiu S Korkolopoulou P Kapsogeorgou EK Kavantzas N Ziakas P. Rates of infiltration by macrophages and dendritic cells and expression of interleukin-18 and interleukin-12 in the chronic inflammatory lesions of sjögren's syndrome: correlation with certain features of immune hyperactivity and factors associated with high risk of lymphoma development. Arthritis Rheumatol (2007) 56(12):3977–88. doi: 10.1002/art.23073 18050195

[127] Izutsu KT Sullivan KM Schubert MM Truelove EL Shulman HM Sale GE. Disordered salivary immunoglobulin secretion and sodium transport in human chronic graft-versus-host disease. Transplantation. (1983) 35(5):441–6. doi: 10.1097/00007890-198305000-00010 6342224

[128] Crossland RE Perutelli F Bogunia-Kubik K Mooney N Milutin Gašperov N Pučić-Baković M. Potential novel biomarkers in chronic graft-Versus-Host disease. Front Immunol (2020) 11. doi: 10.3389/fimmu.2020.602547 PMC7786047 33424849

[129] Yamamoto A Kambara Y Fujiwara H. Impact of oral microbiota on pathophysiology of GVHD. Front Immunol (2023) 14:1132983. doi: 10.3389/fimmu.2023.1132983 36969182 PMC10033631

[130] Pukhalskaya T Smoller BR Becker M Maly A Zadik Y Elad S. Oral white lesion in patients post-hematopoietic stem cell transplantation: a case series demonstrating the diagnostic dilemma. Support Care Cancer. (2021) 29(12):7999–8007. doi: 10.1007/s00520-021-06392-6 34218349

[131] Kuzmina Z Gounden V Curtis L Avila D Rnp TT Baruffaldi J. Clinical significance of autoantibodies in a large cohort of patients with chronic graft-versus-host disease defined by NIH criteria. Am J Hematol (2015) 90(2):114–9. doi: 10.1002/ajh.23885 PMC5535796 25363867

[132] Khan FM Sy S Louie P Ugarte-Torres A Berka N Sinclair GD. Genomic instability after allogeneic hematopoietic cell transplantation is frequent in oral mucosa, particularly in patients with a history of chronic graft-versus-host disease, and rare in nasal mucosa. Blood. (2010) 116(10):1803–6. doi: 10.1182/blood-2009-10-249201 20548092

[133] Hanna GJ Kofman ER Shazib MA Woo SB Reardon B Treister NS. Integrated genomic characterization of oral carcinomas in post-hematopoietic stem cell transplantation survivors. Oral Oncol (2018) 81:1–9. doi: 10.1016/j.oraloncology.2018.04.007 29884406

[134] Fantozzi PJ Villa A Antin JH Treister N. Regression of oral proliferative leukoplakia following initiation of ibrutinib therapy in two allogeneic hematopoietic stem cell transplant recipients. Bone Marrow Transplant. (2020) 55(9):1844–6. doi: 10.1038/s41409-020-0889-2 32273587

[135] Torres-Pereira C Funke V Giovanini AF Lemos CA Jr. Amenabar JM Piazzetta CM. Oral proliferative verrucous leukoplakia (PVL) in a post-bone marrow transplant patient. Biol Blood Marrow Transplant. (2008) 14(10):1197–9. doi: 10.1016/j.bbmt.2008.07.012 18804051

[136] Gomes N Huynh A Borel C Guenounou S Recher C Barres B. Oral acute graft versus host disease after donor lymphocyte infusions: clinicopathological characterization in a monocenter cohort. Oral Oncol (2021) 114:105082. doi: 10.1016/j.oraloncology.2020.105082 33187825

[137] Demarosi F Soligo D Lodi G Moneghini L Sardella A Carrassi A. Squamous cell carcinoma of the oral cavity associated with graft versus host disease: report of a case and review of the literature. Oral Surg Oral Med Oral Pathol Oral Radiol Endod. (2005) 100(1):63–9. doi: 10.1016/j.tripleo.2004.12.008 15953918

[138] Villa A Menon RS Kerr AR De Abreu Alves F. Proliferative leukoplakia: proposed new clinical diagnostic criteria. Oral Dis (2018) 24(5):749–60. doi: 10.1111/odi.12830 29337414

[139] Fraser CJ Bhatia S Ness K Carter A Francisco L Arora M. Impact of chronic graft-versus-host disease on the health status of hematopoietic cell transplantation survivors: a report from the bone marrow transplant survivor study. Blood (2006) 108(8):2867–73. doi: 10.1182/blood-2006-02-003954 PMC1895593 16788100

[140] Stolze J Boor M Hazenberg MD Brand HS Raber-Durlacher JE Laheij A. Oral health-related quality of life of patients with oral chronic graft-versus-host disease. Support Care Cancer (2021) 29(11):6353–60. doi: 10.1007/s00520-021-06197-7 PMC8464572 33884507

[141] González-Moles M Ruiz-Ávila I González-Ruiz L Ayén Á Gil-Montoya JA Ramos-García P. Malignant transformation risk of oral lichen planus: a systematic review and comprehensive meta-analysis. Oral Oncol (2019) 96:121–30. doi: 10.1016/j.oraloncology.2019.07.012 31422203

[142] Ramos-García P González-Moles M. Malignant transformation of oral proliferative verrucous leukoplakia: a systematic review and meta-analysis. Oral Dis (2021) 27(8):1896–907. doi: 10.1111/odi.13831 34009718

[143] Aguirre-Urizar JM Lafuente-Ibáñez de Mendoza I. Malignant transformation of oral leukoplakia: systematic review and meta-analysis of the last 5 years. Oral Dis (2021) 27(8):1881–95. doi: 10.1111/odi.13810 33606345

[144] Lorenzo-Pouso AI Lafuente-Ibáñez de Mendoza I. Critical update, systematic review, and meta-analysis of oral erythroplakia as an oral potentially malignant disorder. J Oral Pathol Med (2022) 51(7):585–93. doi: 10.1111/jop.13304 PMC9545979 35488780

[145] Lodi G Scully C Carrozzo M Griffiths M Sugerman PB Thongprasom K. Current controversies in oral lichen planus: report of an international consensus meeting. part 2. clinical management and malignant transformation. Oral Surg Oral Med Oral Pathol Oral Radiol Endod (2005) 100(2):164–78. doi: 10.1016/j.tripleo.2004.06.076 16037774

[146] Leuci S Coppola N. Oral dysplastic complications after HSCT: single case series of multidisciplinary evaluation of 80 patients. Life (Basel) (2020) 10(10):236. doi: 10.3390/life10100236 33050268 PMC7600275

[147] Mawardi H Elad S Correa ME Stevenson K Woo SB Almazrooa S. Oral epithelial dysplasia and squamous cell carcinoma following allogeneic hematopoietic stem cell transplantation: clinical presentation and treatment outcomes. Bone Marrow Transplant. (2011) 46(6):884–91. doi: 10.1038/bmt.2011.77 PMC3111881 21460866

[148] Santarone S Natale A Angelini S. Secondary oral cancer following hematopoietic cell transplantation. Bone Marrow Transplant (2021) 56(5):1038–46. doi: 10.1038/s41409-020-01147-z 33235350

[149] Hasegawa W Pond GR Rifkind JT Messner HA Lau A Daly AS. Long-term follow-up of secondary malignancies in adults after allogeneic bone marrow transplantation. Bone Marrow Transplant (2005) 35(1):51–5. doi: 10.1038/sj.bmt.1704706 15516939

[150] Chaulagain CP Sprague KA. Clinicopathologic characteristics of secondary squamous cell carcinoma of head and neck in survivors of allogeneic hematopoietic stem cell transplantation for hematologic malignancies. Bone Marrow Transplant (2019) 54(4):560–6. doi: 10.1038/s41409-018-0299-x PMC6484708 30127467

[151] Chen MH Chang PM Li WY Hsiao LT Hong YC Liu CY. High incidence of oral squamous cell carcinoma independent of HPV infection after allogeneic hematopoietic SCT in Taiwan. Bone Marrow Transplant. (2011) 46(4):567–72. doi: 10.1038/bmt.2010.163 20622906

[152] Hernández-Molina G Kostov B Brito-Zerón P Vissink A Mandl T Hinrichs AC. Characterization and outcomes of 414 patients with primary SS who developed hematological malignancies. Rheumatology (Oxford) (2022) 62(1):243–55. doi: 10.1093/rheumatology/keac205 35385104

[153] Kruse AL Grätz KW. Oral carcinoma after hematopoietic stem cell transplantation–a new classification based on a literature review over 30 years. Head Neck Oncol (2009) 1:29. doi: 10.1186/1758-3284-1-29 19624855 PMC2724375

[154] Barba-Montero C Lorenzo-Pouso AI Gándara-Vila P Blanco-Carrión A Marichalar-Mendía X García-García A. Lichenoid areas may arise in early stages of proliferative verrucous leukoplakia: a long-term study of 34 patients. J Oral Pathol Med (2022) 51(6):573–81. doi: 10.1111/jop.13317 PMC9541998 35596256

[155] Alabdulaaly L Villa A Chen T Kerr A Ross N Abreu Alves F. Characterization of initial/early histologic features of proliferative leukoplakia and correlation with malignant transformation: a multicenter study. Mod Pathol (2022) 35(8):1034–44. doi: 10.1038/s41379-022-01021-x 35184151

[156] Thompson LDR Fitzpatrick SG Müller S Eisenberg E Upadhyaya JD Lingen MW. Proliferative verrucous leukoplakia: an expert consensus guideline for standardized assessment and reporting. Head Neck Pathol (2021) 15(2):572–87. doi: 10.1007/s12105-020-01262-9 PMC8134585 33415517

[157] Garcia-Pola MJ Llorente-Pendás S González-Garcia M García-Martín JM. The development of proliferative verrucous leukoplakia in oral lichen planus. a preliminary study. Med Oral Patol Oral Cir Bucal. (2016) 21(3):e328–34. doi: 10.4317/medoral.20832 PMC4867206 27031060

[158] McCarthy C Kazmi A Austin T Ho MW Chengot P Rajlawat BP. The development of proliferative verrucous leukoplakia on a background of oral lichen planus: a case series. Adv Oral Maxillofac Surgery. (2022) 6:100263. doi: 10.1016/j.adoms.2022.100263

[159] McParland H Warnakulasuriya S. Lichenoid morphology could be an early feature of oral proliferative verrucous leukoplakia. J Oral Pathol Med (2021) 50(2):229–35. doi: 10.1111/jop.13129 33185900

[160] Atsuta Y Suzuki R Yamashita T Fukuda T Miyamura K Taniguchi S. Continuing increased risk of oral/esophageal cancer after allogeneic hematopoietic stem cell transplantation in adults in association with chronic graft-versus-host disease. Ann Oncol (2014) 25(2):435–41. doi: 10.1093/annonc/mdt558 24399081

[161] Douglas CM Jethwa AR Hasan W Liu A Gilbert R Goldstein D. Long-term survival of head and neck squamous cell carcinoma after bone marrow transplant. Head Neck (2020) 42(11):3389–95. doi: 10.1002/hed.26402 32820585

[162] Kolb HJ Socié G Duell T Van Lint MT Tichelli A Apperley JF. Malignant neoplasms in long-term survivors of bone marrow transplantation. late effects working party of the European cooperative group for blood and marrow transplantation and the European late effect project group. Ann Intern Med (1999) 131(10):738–44. doi: 10.7326/0003-4819-131-10-199911160-00004 10577296

[163] Lee SJ Flowers ME. Recognizing and managing chronic graft-versus-host disease. Hematol Am Soc Hematol Educ Program (2008), 134–41. doi: 10.1182/asheducation-2008.1.134 19074071

[164] Saidu NEB Bonini C Dickinson A Grce M Inngjerdingen M Koehl U. New approaches for the treatment of chronic graft-Versus-Host disease: current status and future directions. Front Immunol (2020) 11:578314–. doi: 10.3389/fimmu.2020.578314 PMC7583636 33162993

[165] Wolff D Fatobene G Rocha V Kröger N Flowers ME. Steroid-refractory chronic graft-versus-host disease: treatment options and patient management. Bone Marrow Transplant. (2021) 56(9):2079–87. doi: 10.1038/s41409-021-01389-5 PMC8410585 34218265

[166] Gooptu M Antin JH. GVHD prophylaxis 2020. Front Immunol (2021) 12:605726. doi: 10.3389/fimmu.2021.605726 33897681 PMC8059368

[167] Cutler C Kim HT Bindra B Sarantopoulos S Ho VT Chen YB. Rituximab prophylaxis prevents corticosteroid-requiring chronic GVHD after allogeneic peripheral blood stem cell transplantation: results of a phase 2 trial. Blood. (2013) 122(8):1510–7. doi: 10.1182/blood-2013-04-495895 PMC3750344 23861248

[168] Afram G Watz E Remberger M Nygell UA Sundin M Hägglund H. Higher response rates in patients with severe chronic skin graft-versus-host disease treated with extracorporeal photopheresis. Central-European J Immunol (2019) 44(1):84–91. doi: 10.5114/ceji.2018.75831 PMC6526584 31114441

[169] Zeiser R Lee SJ. Three US food and drug administration-approved therapies for chronic GVHD. Blood (2022) 139(11):1642–5. doi: 10.1182/blood.2021014448 PMC8931512 35081254

[170] Didona D Caposiena Caro RD Sequeira Santos AM Solimani F Hertl M. Therapeutic strategies for oral lichen planus: state of the art and new insights. Front Med (2022) 9. doi: 10.3389/fmed.2022.997190 PMC9578567 36267615

[171] Kaurinovic M Delli K. Effect of ruxolitinib on the oral mucosa of patients with steroid-refractory chronic graft-versus-Host disease and oral involvement. Clin Oral Investig (2022) 26(5):4209–16. doi: 10.1007/s00784-022-04393-1 PMC9072523 35169886

[172] Sava A Piciu A Pasca S Mester A Tomuleasa C. Topical corticosteroids a viable solution for oral graft versus host disease? A systematic insight on randomized clinical trials. Medicina (Kaunas) (2020) 56(7):349. doi: 10.3390/medicina56070349 32674447 PMC7404764

[173] Mawardi H Stevenson K Gokani B Soiffer R Treister N. Combined topical dexamethasone/tacrolimus therapy for management of oral chronic GVHD. Bone Marrow Transplant. (2010) 45(6):1062–7. doi: 10.1038/bmt.2009.301 19881552

[174] Im A Pusic I Onstad L Kitko CL Hamilton BK Alousi AM. Patient-reported treatment response in chronic graft-versus-host disease. Haematologica (2023). doi: 10.3324/haematol.2023.282734 PMC10772515 37226713

[175] Curtis RE Metayer C Rizzo JD Socie G Sobocinski KA Flowers ME. Impact of chronic GVHD therapy on the development of squamous-cell cancers after hematopoietic stem-cell transplantation: an international case-control study. Blood. (2005) 105(10):3802–11. doi: 10.1182/blood-2004-09-3411 PMC1895092 15687239

[176] Agha-Hosseini F Mirzaii-Dizgah I Ghavamzadeh L Ghavamzadeh A Tohidast-Acrad Z. Effect of pilocarpine hydrochloride on unstimulated whole saliva flow rate and composition in patients with chronic graft-versus-host disease (cGVHD). Bone Marrow Transplant. (2007) 39(7):431–4. doi: 10.1038/sj.bmt.1705621 17310130

[177] Zadik Y Zeevi I Luboshitz-Shon N Dakwar N Wolff A Shapira MY. Safety and efficacy of an intra-oral electrostimulator for the relief of dry mouth in patients with chronic graft versus host disease: case series. Med Oral Patol Oral Cir Bucal. (2014) 19(3):e212–9. doi: 10.4317/medoral.19429 PMC4048107 24121920

[178] Balasubramaniam R Alawi F DeRossi S. Superficial mucoceles in chronic graft-versus-host disease: a case report and review of the literature. Gen Dent. (2009) 57(1):82–8. 19146147

[179] Fantozzi PJ Treister N Shekar R Woo SB Villa A. Intralesional triamcinolone acetonide therapy for inflammatory oral ulcers. Oral surgery Oral medicine Oral Pathol Oral radiology. (2019) 128(5):485–90. doi: 10.1016/j.oooo.2019.07.024 31466871

[180] Epstein JB Nantel S Sheoltch SM. Topical azathioprine in the combined treatment of chronic oral graft-versus-host disease. Bone Marrow Transplant. (2000) 25(6):683–7. doi: 10.1038/sj.bmt.1702192 10734307

[181] Wolff D Anders V Corio R Horn T Morison WL Farmer E. Oral PUVA and topical steroids for treatment of oral manifestations of chronic graft-vs.-host disease. Photodermatol Photoimmunol Photomed (2004) 20(4):184–90. doi: 10.1111/j.1600-0781.2004.00102.x 15238096

[182] Epstein JB Raber-Durlacher JE Epstein GL Hazenberg MD Tzachanis D Spielberger RT. Chronic oral graft-versus-host disease: induction and maintenance therapy with photobiomodulation therapy. Support Care Cancer. (2021) 29(3):1387–94. doi: 10.1007/s00520-020-05626-3 32666212

[183] Epstein JB Raber-Durlacher JE Lill M Linhares YP Chang J Barasch A. Photobiomodulation therapy in the management of chronic oral graft-versus-host disease. Support Care Cancer. (2017) 25(2):357–64. doi: 10.1007/s00520-016-3401-1 27655559

[184] Garming-Legert K Tour G Sugars R von Bahr L Davies LC Le Blanc K. Enhanced oral healing following local mesenchymal stromal cell therapy. Oral Oncol (2015) 51(12):e97–9. doi: 10.1016/j.oraloncology.2015.09.008 26428076

[185] González-Moles M Ramos-García P. A scoping review on gaps in the diagnostic criteria for proliferative verrucous leukoplakia: a conceptual proposal and diagnostic evidence-based criteria. Cancers (Basel) (2021) 13(15):3669. doi: 10.3390/cancers13153669 34359571 PMC8345058

[186] Devic I Shi M Schubert MM Lloid M Izutsu KT Pan C. Proteomic analysis of saliva from patients with oral chronic graft-versus-host disease. Biol Blood marrow Transplant (2014) 20(7):1048–55. doi: 10.1016/j.bbmt.2014.03.031 PMC4552451 24704387

[187] Tamma R Limongelli L Maiorano E Pastore D Cascardi E Tempesta A. Vascular density and inflammatory infiltrate in primary oral squamous cell carcinoma and after allogeneic hematopoietic stem cell transplantation. Ann Hematol (2019) 98(4):979–86. doi: 10.1007/s00277-018-3575-3 30519712

[188] Atri C Guerfali FZ. Role of human macrophage polarization in inflammation during infectious diseases. Int J Mol Sci (2018) 19(6):1801. doi: 10.3390/ijms19061801 29921749 PMC6032107

[189] Tuzlak S Dejean AS Iannacone M Quintana FJ Waisman A Ginhoux F. Repositioning TH cell polarization from single cytokines to complex help. Nat Immunol (2021) 22(10):1210–7. doi: 10.1038/s41590-021-01009-w 34545250

